# The median and the mode as robust meta‐analysis estimators in the presence of small‐study effects and outliers

**DOI:** 10.1002/jrsm.1402

**Published:** 2020-03-10

**Authors:** Fernando P. Hartwig, George Davey Smith, Amand F. Schmidt, Jonathan A. C. Sterne, Julian P. T. Higgins, Jack Bowden

**Affiliations:** ^1^ Postgraduate Program in Epidemiology Federal University of Pelotas Pelotas Brazil; ^2^ MRC Integrative Epidemiology Unit University of Bristol Bristol UK; ^3^ Population Health Sciences University of Bristol Bristol UK; ^4^ Institute of Cardiovascular Science, Faculty of Population Health University College London London UK; ^5^ Faculty of Science and Engineering, Groningen Research Institute of Pharmacy University of Groningen Groningen The Netherlands; ^6^ University of Exeter College of Medicine and Health Exeter UK

**Keywords:** median, meta‐analysis, mode, robust estimation, small‐study effects

## Abstract

Meta‐analyses based on systematic literature reviews are commonly used to obtain a quantitative summary of the available evidence on a given topic. However, the reliability of any meta‐analysis is constrained by that of its constituent studies. One major limitation is the possibility of small‐study effects, when estimates from smaller and larger studies differ systematically. Small‐study effects may result from reporting biases (ie, publication bias), from inadequacies of the included studies that are related to study size, or from reasons unrelated to bias. We propose two estimators based on the median and mode to increase the reliability of findings in a meta‐analysis by mitigating the influence of small‐study effects. By re‐examining data from published meta‐analyses and by conducting a simulation study, we show that these estimators offer robustness to a range of plausible bias mechanisms, without making explicit modelling assumptions. They are also robust to outlying studies without explicitly removing such studies from the analysis. When meta‐analyses are suspected to be at risk of bias because of small‐study effects, we recommend reporting the mean, median and modal pooled estimates.

## 1 INTRODUCTION

Meta‐analysis is used to obtain a quantitative summary of the evidence from multiple studies on a given topic and is often undertaken as part of a systematic review.^1^, ^2^ In its archetypal form, meta‐analysis provides an overall effect estimate for a well‐defined intervention that has been assessed across several independent clinical trials, although it can also be applied to other study designs. Meta‐analyses also provide an opportunity to explore between‐study heterogeneity, which might highlight possible explanations for variation in treatment effects.^1^, ^2^

Systematic differences between effect estimates from different studies may also indicate the presence of bias, which we wish to understand if possible and, ultimately, seek to remove from the analysis. Such differences may be due to flaws or limitations in the design, conduct or analysis of the included studies: for example, the failure of some randomized trials to conceal the allocation sequence from those recruiting participants, or the use of inappropriate imputation methods for missing endpoint data. The seriousness of these types of limitations may be associated with the size of the study, leading to a type of heterogeneity in which estimates from larger and smaller studies differ systematically.

A further threat to the validity of meta‐analyses is publication bias,^2^ when the probability that results are reported and published is related to the direction or magnitude of their findings,^3^ so that published results are a biased sample of all results generated. This bias is less likely to affect larger than smaller studies, due to a combination of pressure to publish by external funders or collaborators, the greater inherent publishing appeal of larger studies, and because an increased sample size raises the likelihood of achieving conventional statistical significance when the true treatment effect is nonzero.^2^

It is not necessarily the case that, in the presence of systematic differences associated with study size, smaller studies are less reliable than larger ones Systematic differences between large and small studies may be to reasons other than bias: for example, if the intervention was implemented more effectively in the smaller studies.^4^ Therefore, the phenomenon where the reported treatment effect is associated with study size in a meta‐analysis encompasses many different mechanisms and is referred to with the umbrella‐term “small‐study effects.”^5^ It is difficult to identify whether an association between study size and reported treatment effect is due to true heterogeneity, biases in the results of individual studies, selective reporting (or publication), or a combination of these.^2^, ^6^

Many methods to detect and correct for small‐study effects have been proposed. One of the earliest of such methods is the funnel plot (where study‐specific point estimates are plotted against their precision), which was proposed more than 30 years ago.^7^ Difficulties in visual interpretation of funnel plots motivated the development of tests for funnel plot asymmetry^4^, ^8^ and approaches that “correct” for asymmetry, such as regression and trim‐and‐fill estimators.^9^, ^10^, ^11^ However, these approaches make either implicit or explicit assumptions about the asymmetry‐generating process so that their performance suffers when the true bias mechanism differs from that assumed.

Here, we propose two simple estimators that are robust to small‐study effects, while making no assumptions about their precise nature. They were originally proposed for causal inference in summary data Mendelian randomization.^12^, ^13^ From a statistical perspective, this technique has strong parallels with meta‐analysis.^14^, ^15^

## 2 META‐ANALYSIS DATASETS

**Figure 1:**
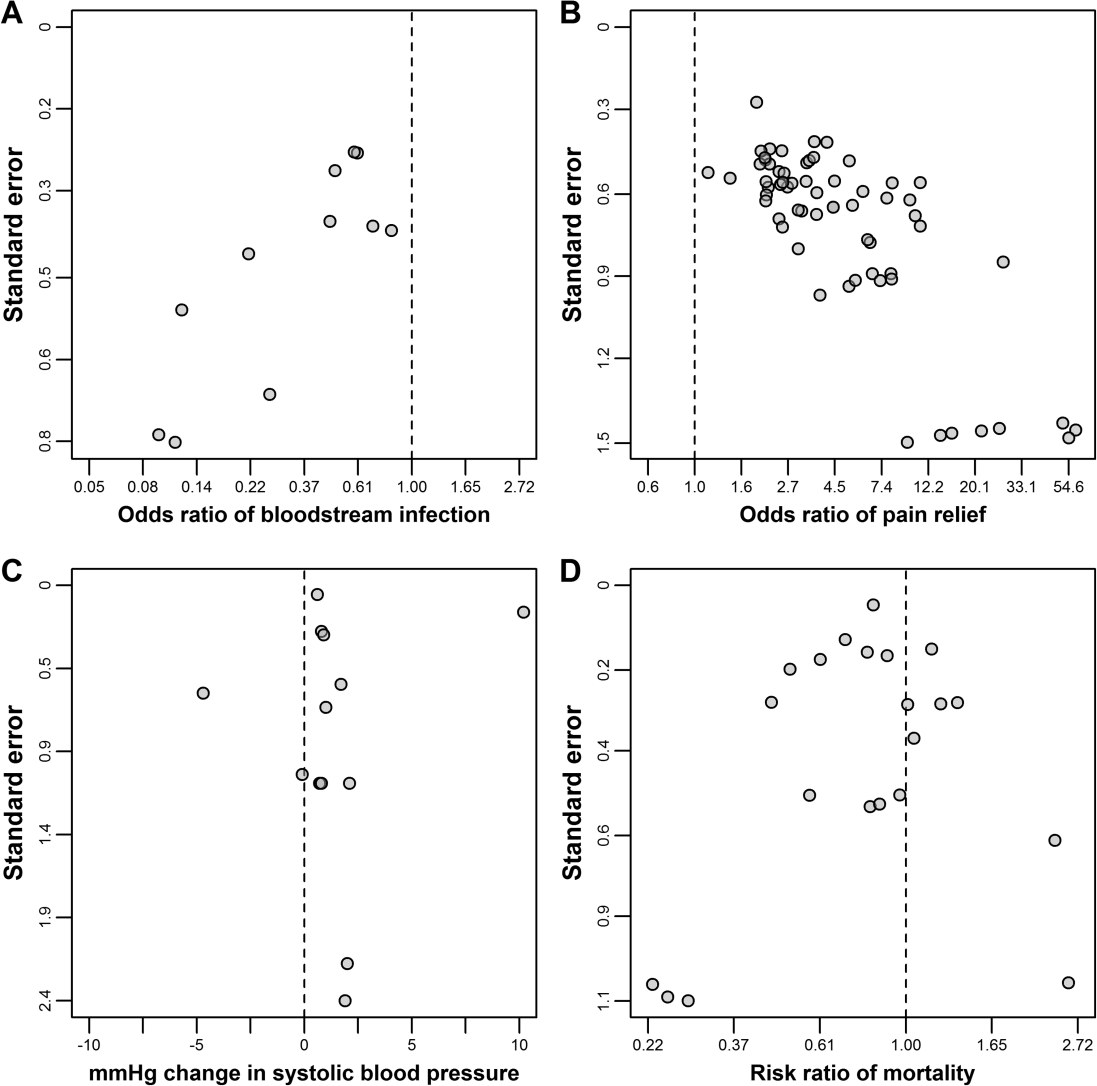
Funnel plots of the catheter (panel A), aspirin (panel B), sodium (panel C), and streptokinase (panel D) meta‐analyses

Before presenting the estimators, we describe four meta‐analysis datasets that will be used throughout the article to explain the proposed estimators and illustrate their application. In addition to funnel plots (Figure 1), we characterize these datasets using the following statistics: (a) Asymmetry, which we defined as the Egger test's coefficient (*γ*), that is, the slope of an inverse variance weighted linear regression of effect estimates on standard errors.^4^ *P*‐values were calculated using *t*‐test with *K* − 2 degrees of freedom, where *K* is the number of studies; and (b) Between‐study inconsistency, defined as the conventional *I*^2^ statistic. Importantly, the *I*^2^ statistic does not quantify variation in the true effect sizes across studies, but rather statistical inconsistencies in the results of the studies.^16^ For example, for a given data‐generating mechanism producing a given amount of variation in the true effect sizes (ie, heterogeneity), increasing the size of the studies will generally increase *I*^2^ (because the study‐specific confidence intervals will get narrower, thus increasing the statistical power to detect inconsistencies).

- Catheter dataset (Figure 1A): this meta‐analysis, originally conducted by Veenstra et al,^17^ evaluated 11 trials comparing chlorhexidine‐silver sulfadiazine‐impregnated vs nonimpregnated catheters with regard to risk of catheter‐related bloodstream infection. These data presented a large correlation between effect estimates and their precision (*γ*=3.05 [*P*‐value = 0.007]) (which translates into substantial asymmetry on the funnel plot), and high between‐study inconsistency (*I*^2^ = 60%).
- Aspirin dataset (Figure 1B): this meta‐analysis, originally conducted by Edwards et al,^18^ evaluated 63 trials investigating the effect of a single dose of oral aspirin on pain relief (50% reduction in pain). Asymmetry was also strong in magnitude (*γ* = 2.11 [*P*‐value = 5.2 × 10^−9^]), but there was low between‐study inconsistency (*I*^2^ = 10%).
- Sodium dataset (Figure 1C): this meta‐analysis was originally conducted by Leyvraz et al,^19^ and assessed the effect of sodium intake on blood pressure in children and adolescents. We focused on the meta‐analysis of 13 experimental studies (three of which were not randomized trials) of systolic blood pressure. Asymmetry was strong in magnitude (*γ* = 2.60), but there was no strong statistical evidence against the null hypothesis of no asymmetry (*P*‐value = 0.679). Moreover, there was high between‐study inconsistency (*I*^2^ = 99%). As shown in Section 4, both *γ* and *I*^2^ are substantially attenuated upon removal of two studies classified as influential by Leyvraz et al.
- Streptokinase dataset (Figure 1D): this meta‐analysis, originally conducted by Yusuf et al^20^ and updated by Egger et al,^4^ includes 21 trials evaluating the effect of streptokinase therapy on mortality risk. These data presented moderate inconsistency (*I*^2^ = 34%), but very little evidence of asymmetry (*γ*= − 0.06, *P*‐value = 0.868). Given that in this dataset there is no strong indication of small‐study effects, these data were used as a positive control, where all estimators are expected to give similar answers.

## 3 METHODS

We now give a nontechnical explanation of our proposed estimators to motivate their utility. We then provide a more technical description of our approach, by first describing the assumed data generating mechanism and the proposed estimation procedures.

### 3.1 Nontechnical intuition

The standard way to combine studies in a meta‐analysis is via a weighted mean of study‐specific results, where the weight given to each study estimate is the inverse of its variance (thus reflecting its precision). Under the assumption that all included studies provide valid estimates of the same underlying treatment effect, this “fixed effect” approach provides the summary estimate that is the most efficient, that is, most precise and therefore with the highest power to detect a nonzero treatment effect.

Skewness affects the utility of the mean as a measure of central tendency. For example, the distribution of income is typically positively skewed due to the presence of a few individuals who are much wealthier than most of the population. In such cases, statistics such as the median or the mode are often used instead of the mean as central tendency measures to quantify “typical” income, although for some applications the mean will still be the statistic of interest.

Skewness in individual participant datasets is analogous to funnel plot asymmetry in meta‐analyses. Examples of funnel plot asymmetry are shown in Figure 1A,B. In the aspirin dataset (Figure 1B), smaller studies have generally larger point estimates. Given that the mean is more sensitive to asymmetry than the median and the mode (and the same for their inverse variance weighted versions, described in Section 3.3), estimates obtained using the latter two measures would be closer to the bulk of evidence in the meta‐analysis. In cases where the strong asymmetry makes it implausible to discard the possibility of bias, estimators that yield combined estimates closer to the bulk of the funnel plot are likely to be more reliable.

A second situation where the mean may not be a useful central tendency statistic is when there are outliers. Using again the example of income, the mean income of a population will be largely influenced by the extreme wealth of a tiny proportion of individuals and will not reflect the typical income of the majority. Again, the median or the mode provide a central tendency statistic that is closer to most data points than the mean. The presence of a few outliers in a large population may not be problematic in typical studies using individual participant data because their influence is diluted. However, a meta‐analysis often contains a small number of data points (study results), increasing the relative influence of outliers on the combined estimate. For example, in the sodium dataset (Figure 1C), Leyvraz et al^19^, using a statistical criterion, classified two studies as outliers. In the Results section, we show that these two studies have a substantial influence in the results by pushing the weighted mean, but neither the median nor the mode, away from the bulk of the funnel plot.

We now provide a more formal justification for using the median or mode in the meta‐analysis context to achieve robustness to small‐study effects and outlying studies, focusing on small‐study effects. We return to the topic of outlying studies when analyzing real meta‐analysis datasets.

### 3.2 Data generating mechanism

We first define a summary data generating mechanism with *K* studies indexed by *j* (*j* = 1, 2, …, *K*) in a form that allows us to incorporate different types of small‐study effects (Box 1). We assume each study reports an estimated mean difference between groups (eg, an experimental intervention and a standard intervention) denoted by ${\hat{\beta }}_{j}$, where:

(1) $${\hat{\beta }}_{j}=\beta +b_{j}+\sigma_{j}\varepsilon_{j}.$$

Here:

- *β* is the average effect of the experimental compared with standard intervention on the outcome;
- *b*_*j*_ denotes the bias/heterogeneity parameter for study *j*;
- *σ*_*j*_ is the standard error of the mean difference;
- *ε*_*j*_~*N*(0, 1, *l*_*j*_, *u*_*j*_) is drawn from a standard truncated normal distribution with lower limit *l*_*j*_ and upper limit *u*_*j*_;
- The parameters *b*_*j*_, *l*_*j*_, and *u*_*j*_ are all allowed to depend on the study size, *n*_*j*_.

**Box 1. General principles of our small‐study effects models**

We use model (1) to explore two types of small‐study effects: bias due to systematic differences between small and large studies due to study quality (type (a)), and bias due to the specific environment of selective reporting and publication in operation at the time when study *j* was conducted (type (b)).

For type (a), we assume that differences between small and large published studies are due to fundamental properties of each study that are correlated with study size (*n*_*j*_). For this, the fixed bias parameter *b*_*j*_ is a function of *n*_*j*_ such that:

0 ≤ *b*_*j*_ ≤ *b*_*k*_ or 0 ≥ *b*_*j*_ ≥ *b*_*k*_, whenever *n*_*k*_ ≤ *n*_*j*_.

We will investigate cases where the bias disappears only asymptotically as a study size grows infinitely large, and cases where the bias disappears beyond a threshold study size, *N*. That is:

*b*_*j*_ → 0 as *n*_*j*_ → ∞, or *b*_*j*_ = 0 if *n*_*j*_ ≥ *N* for some (large) *N*.

Type (b) bias is not a fundamental component of the study itself, but instead the result of selective reporting and publication (ie, publication bias). We induce this through the random error component of model (1), *ε*_*j*_, by defining *l*_*j*_ or *u*_*j*_ as functions of *n*_*j*_ such that, whenever *n*_*k*_ ≤ *n*_*j*_:

*l*_*j*_ ≤ *l*_*k*_ ≤ 0, and therefore 0 ≤ *E*[*ε*_*j*_| *n*_*j*_] ≤ *E*[*ε*_*k*_| *n*_*k*_]; or

*u*_*j*_ ≥ *u*_*k*_ ≥ 0, and therefore 0 ≥ *E*[*ε*_*j*_| *n*_*j*_] ≥ *E*[*ε*_*k*_| *n*_*k*_].

For example, assume that type (b) bias is always positive, so that and *E*[*ε*_*j*_| *n*_*j*_] ≥ 0. This corresponds to a situation where the selection process favors the publication of studies that reported positive effect estimates. This could be achieved defining *l*_*j*_ as a nonincreasing function of *n*_*j*_.

Similar to the type (a) bias model, we will explore cases where:

*l*_*j*_ →  − ∞ and *u*_*j*_ →  ∞  ⇒ *E*[*ε*_*j*_| *n*_*j*_] → 0 as *n*_*j*_ → ∞; or

*l*_*j*_ =  − ∞ and *u*_*j*_ =  ∞  ⇒ *E*[*ε*_*j*_| *n*_*j*_] = 0 if *n*_*j*_ ≥ *N* for some large *N*.

An important distinction between type (a) and type (b) bias is their respective effect on the variance of the study‐specific estimates. Type (a) bias will generally increase their variability, leading to over‐dispersion, or heterogeneity. Type (b) bias, by contrast, can have the opposite effect of reducing their variability, because of the truncation in the distribution of *ε*_*j*_. That is, in the presence of this bias, Var[*ε*_*j*_| *n*_*j*_] will generally be less than 1, and Var[*ε*_*j*_| *n*_*j*_] ≥ Var[*ε*_*k*_| *n*_*k*_] whenever *n*_*k*_ ≤ *n*_*j*_.This phenomenon leads to under‐dispersion across the set of study‐specific estimates constituting the meta‐analysis.

Standard meta‐analysis models correspond to *l* =  − ∞ and *u* = ∞, in which case *ε*_*j*_ denotes random error due to sampling variation. A conventional fixed‐effects model would correspond to *b*_*j*_ = 0 for all studies, and a random‐effects model to *b*_*j*_~*N*(0, *τ*^2^). This conventional random‐effects distribution allows for between‐study differences due to biases or due to other sources of heterogeneity; often it is not possible to distinguish one from the other.

Throughout this article we assume a fixed treatment effect (as assumed by our proposed estimators, which do not explicitly model between‐study heterogeneity), so that nonzero values of *b*_*j*_ occur only due to bias and not to other sources of heterogeneity. Small‐study effects are present if the biases *b*_*j*_ are correlated with study sizes *n*_*j*_. We recognize that not all systematic differences between small and large studies are due to differential bias. Small‐study effects also arise if *b*_*j*_ represents (non‐bias‐related) treatment effect heterogeneity that happens to be correlated with *n*_*j*_. Our methods are not intended to address such situations. We discuss the practical application of the proposed estimators in the presence of heterogeneity in Section 5.

Small‐study effects may also arise due to selective reporting and publication, which can be induced in our model by allowing the truncation limits for *ε*_*j*_ (ie, *l*_*j*_ and *u*_*j*_) to be correlated with *n*_*j*_. In the sections to follow, we will use *b*_*j*_ and the truncation limits for *ε*_*j*_ to induce different types of small‐study effects in the data, as described in Box 1. A general expression for the expected value of study *j*'s effect estimate ${\hat{\beta }}_{j}$, based on *n*_*j*_ participants is:

(2) $$E\left[{\hat{\beta }}_{j}|n_{j}\right]=\beta +b_{j}+\sigma_{j}E\;\left[\varepsilon_{j}|n_{j}\right].$$

### 3.3 Robust central tendency statistics in meta‐analysis

We now introduce three estimators for *β*: the standard weighted mean plus two novel estimators and discuss their ability to return consistent estimates under the assumed data generating mechanism. For the purposes of clarity only, we will assume throughout the remainder of Section 3 that *b*_*j*_ is the sole source of bias in Equation (1), that is, that *E*[*ε*_*j*_|*n*_*j*_] = 0.

#### 3.3.1 The weighted mean

A standard fixed‐effect meta‐analysis estimates the effect size parameter *β* as an inverse‐variance weighted average (or combined mean) of the individual study estimates. That is:

(3) $${\hat{\beta }}_{\mathrm{FE}}=\frac{\sum_{j=1}^{K}{\hat{\beta }}_{j}\sigma_{j}^{-2}}{\sum_{j=1}^{K}\sigma_{j}^{-2}}.$$

If even a single study contributes a biased estimate to the meta‐analysis (eg, via a non‐zero *b*_*j*_), then the combined mean will also be biased (unless the biases in different studies happen to cancel out). That is, using the notation of formula (1):

$E\left[{\hat{\beta }}_{\mathrm{FE}}\right]\neq \beta \;$in general, whenever *b*_*j*_ ≠ 0 for at least one study *j* in 1, …, *K*.

For this reason, in the language of robust statistics, the mean is said to have a 0% “breakdown” level. The exception would be in a situation where *b*_*j*_ is negative for some studies and positive for other studies such that the net bias is zero, that is, $\sum_{j=1}^{K}b_{j}\sigma_{j}^{-2}=0$.

#### 3.3.2 The weighted median

The weighted median^12^ estimate is defined as the 50^th^ percentile of the inverse‐variance weighted empirical distribution of the study specific estimates, which can be calculated as follows. Assume that the ${\hat{\beta }}_{j}$s are sorted in ascending order so that ${\hat{\beta }}_{1}\leq {\hat{\beta }}_{2}\ldots \leq {\hat{\beta }}_{K}$. Let the standardized inverse‐variance weight for study *j* be defined as $w_{j}=\frac{\sigma_{j}^{-2}}{\sum_{j=1}^{K}\sigma_{j}^{-2}}$ and sort them in the same order as the ${\hat{\beta }}_{j}$s. Let $s_{j}=\sum_{g=1}^{j}w_{g}$ denote the sum of standardized weights up to and including the *j*th study. This means that ${\hat{\beta }}_{j}$ is the $q_{j}=100\left(s_{j}-\frac{w_{j}}{2}\right)$th percentile of the weighted empirical distribution of ${\hat{\beta }}_{j}$s.

The weighted median estimate is the 50^th^ percentile of this weighted empirical distribution, so it will be equal to ${\hat{\beta }}_{j}$if *s*_*j*_ = 0.5. In practice, no study lies exactly at the 50^th^ percentile, so this quantity is estimated by linear interpolation between its neighboring estimates ${\hat{\beta }}_{j^{*}}$ and ${\hat{\beta }}_{j^{†}}$, which correspond to the effect estimates reported by the studies located immediately before and after the 50% percentile, respectively (ie, $q_{j^{*}}=\max\left(q_{1},q_{2},\ldots ,q_{j^{*}}\right)\;$, $q_{j^{†}}=\min\left(q_{j^{†}},q_{j^{†}+1},\ldots ,q_{K}\right)\;$, and $q_{j^{*}}<0.5<q_{j^{†}}$). In this case, the weighted median estimate ${\hat{\beta }}_{\mathrm{WM}}$ is:

(4) $${\hat{\beta }}_{\mathrm{WM}}={\hat{\beta }}_{j^{*}}+\left({\hat{\beta }}_{j^{†}}-{\hat{\beta }}_{j^{*}}\right)\frac{0.5-q_{j^{*}}}{q_{j^{†}}-q_{j^{*}}}.$$

The weighted median does not require that all ${\hat{\beta }}_{j}$s are consistent estimates for the true effect *β*. More specifically, provided that both ${\hat{\beta }}_{j^{*}}$ and ${\hat{\beta }}_{j^{†}}$ are consistent for *β*, the ${\hat{\beta }}_{\mathrm{WM}}$ is consistent. This implies that, as the number of studies grows indefinitely large, the ${\hat{\beta }}_{\mathrm{WM}}$ is consistent if up to (but not including) 50% of the total weight in the analysis comes from biased studies, that is, $\left(\sum_{j=1}^{K}I\left(b_{j}>0\right)w_{j}\right)<50\%$. This means that the weighted median has a breakdown level of 50%. Of note, if *b*_*j*_ is negative for some studies and positive for other studies, it is possible that both ${\hat{\beta }}_{j^{*}}$ and ${\hat{\beta }}_{j^{†}}$ are consistent for *β* even if more than 50% of the weight comes from biased studies.

#### 3.3.3 The mode‐based estimate

The mode‐based estimate (MBE)^13^ exploits an assumption we refer to as the zero modal bias assumption (ZEMBA). This states that the most common value of the bias parameter *b*_*j*_ is zero. If ZEMBA holds, the mode of all ${\hat{\beta }}_{j}$s (hereafter referred to as ${\hat{\beta }}_{\mathrm{MBE}}$) is consistent for *β*, even if the majority of ${\hat{\beta }}_{j}$s are biased.

More formally, ${\hat{\beta }}_{\mathrm{MBE}}$ is consistent if *ω*_0_ > max(*ω*_1_, *ω*_2_, …, *ω*_*v*_), where $\omega_{0}=\sum_{j=1}^{K}w_{j}I\left(b_{j}=0\right)$ denotes the sum of weights provided by studies with zero bias, and *ω*_1_, *ω*_2_, and *ω*_*v*_ are the sum of weights provided by studies that have the smallest, the second smallest, and the largest identical bias terms, respectively. For example, suppose that there are 10 studies and *b*_1_ = *b*_2_ < *b*_3_ < *b*_4_ = *b*_5_ = *b*_6_ = 0 < *b*_7_ = *b*_8_ = *b*_9_ < *b*_10_. In this case, $\omega_{0}=\sum_{j\in \left\{4,5,6\right\}}w_{j}\;$, $\omega_{1}=\sum_{j\in \left\{1,2\right\}}w_{j}\;$, *ω*_2_ =  ∑ *w*_3_, $\omega_{3}=\sum_{j\in \left\{7,8,9\right\}}w_{j}$, and *ω*_4_ =  ∑ *w*_10_.

It is possible to exploit ZEMBA in different ways. Here, as in Hartwig et al,^13^ we use the mode of the smoothed, inverse‐variance weighted empirical density function of all ${\hat{\beta }}_{j}$s as the MBE. More specifically, ${\hat{\beta }}_{\mathrm{MBE}}$ is the value of *x* that maximizes *f*(*x*) (ie, $f\left({\hat{\beta }}_{M}\right)=\max\left[f\left(x\right)\right]$). *f*(*x*) is the normal kernel density function:

(5) $$f\left(x\right)=\frac{1}{h\sqrt{2\pi }}\sum_{j=1}^{K}w_{j}\exp\left[-\frac{1}{2}{\left(\frac{x-{\hat{\beta }}_{j}}{h}\right)}^{2}\right],$$

where *h* is the smoothing bandwidth parameter.^21^ This parameter regulates a bias‐variance trade‐off, with smaller values of *h* reducing both bias and precision. Given that the error terms in Equation (1) were drawn from a standard truncated normal distribution, a normal kernel is expected to yield adequate density estimates.

Silverman's rule is commonly used with a normal kernel to calculate *h*. We used the modified Silverman's bandwidth selection rule proposed by Bickel et al^22^, which reduces the influence of outliers compared with the conventional Silverman's rule:

(6) $$h=\frac{0.9\min\left(\mathrm{sd}\left({\hat{\beta }}_{j}\right),1.4826\mathrm{mad}\left({\hat{\beta }}_{j}\right)\right)}{L^{\frac{1}{5}}},$$

where $\mathrm{sd}\left({\hat{\beta }}_{j}\right)$ and $\mathrm{mad}\left({\hat{\beta }}_{j}\right)$, respectively, denote the standard deviation and the median absolute deviation of the median of the study‐level point treatment effect estimates.

The exact breakdown level of the MBE depends on max(*ω*_1_, *ω*_2_, …, *ω*_*v*_), which is unknown. If all biased studies estimate exactly the same effect parameter, then ZEMBA will only be satisfied if up to (but not including) 50% of the weight comes from biased studies. The upper limit of the breakdown level is up to (but not including) 100% and corresponds to the situation where all biased studies estimate different effect parameters. Therefore, the breakdown level of the MBE ranges from 50% to 100%.

#### 3.3.4 Standard errors for the weighted median and the MBE

Standard errors of the weighted median and the MBE can be calculated using parametric bootstrap, which naturally incorporates any between‐study heterogeneity. More specifically, suppose that *R* bootstrap iterations are to be performed. For each iteration *r* ∈ {1, …, *R*}, the bootstrapped point estimate from the *j*th study (${\hat{\beta }}_{j}^{r}$) is sampled from the normal distribution ${\hat{\beta }}_{j}^{r}∼N\left({\hat{\beta }}_{j},\sigma_{j}^{2}\right)$. Then each estimator is applied to the current set ${\left\{{\hat{\beta }}_{j}^{r}\right\}}_{j=1}^{K}$ of resampled point estimates, generating ${\hat{\beta }}_{\mathrm{WM}}^{r}$ and ${\hat{\beta }}_{\mathrm{MBE}}^{r}$. Repeating this step *R* times yields the sets ${\left\{{\hat{\beta }}_{\mathrm{WM}}^{r}\right\}}_{r=1}^{R}$and ${\left\{{\hat{\beta }}_{\mathrm{MBE}}^{r}\right\}}_{r=1}^{R}$, which are empirical sampling distributions of the weighted median and the MBE, respectively. We used a robust standard deviation estimator (the median absolute deviation from the median corrected for asymptotic normal consistency) to calculate the standard deviation from each empirical distribution, which is an estimate of the standard error. Finally, these can be used to calculate confidence intervals based on a normal approximation.

### 3.4 Illustrating the identifying assumptions of the mean, median, and mode

**Figure 2:**
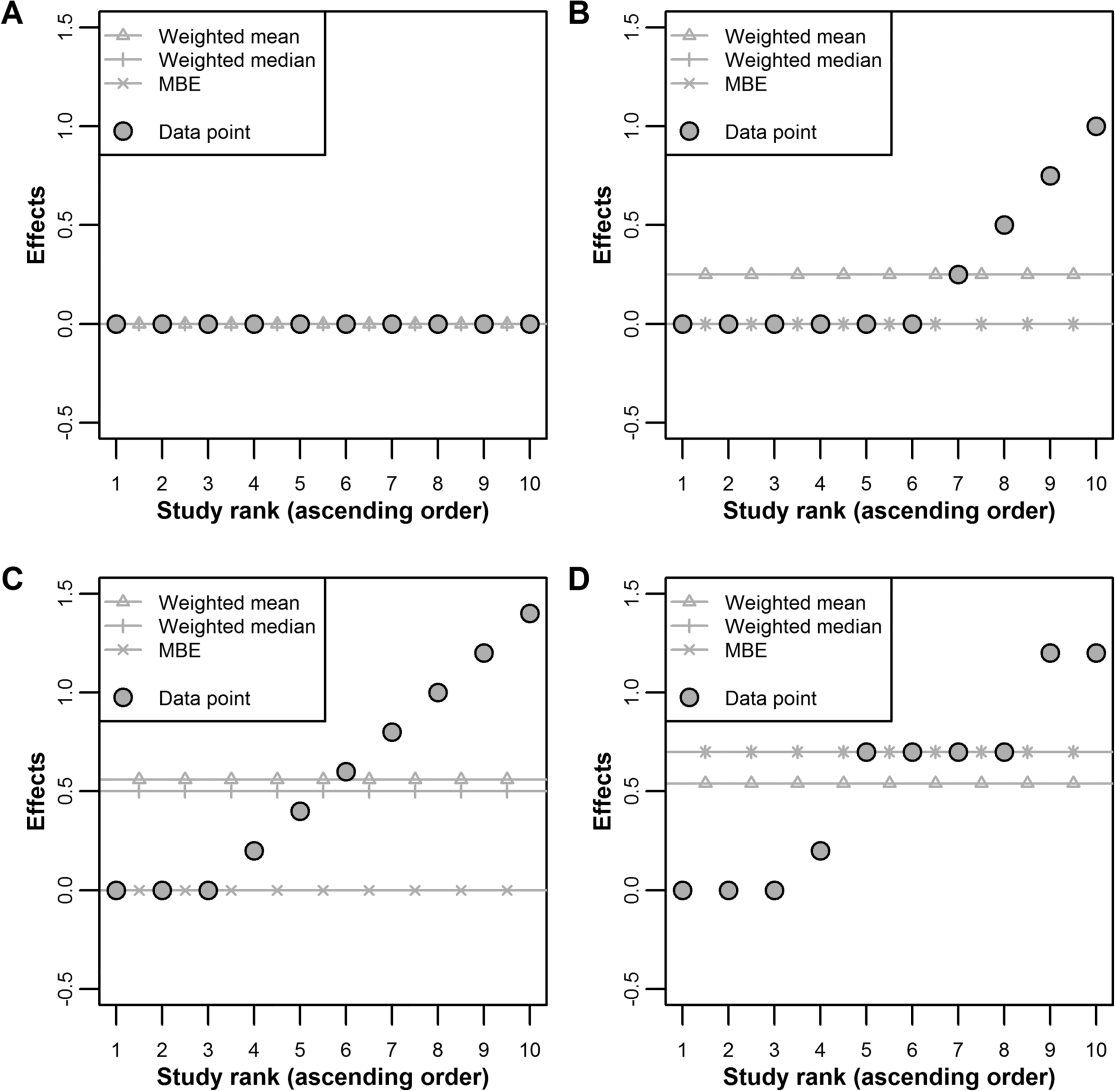
Illustration of the assumptions underlying the weighted mean, weighted median and the mode‐based estimate (MBE) estimators. Studies are assumed to have the same weights in the meta‐analysis, and are sorted in ascending order of point estimate. The true effect is zero. A, No heterogeneity between studies. B, Four out of 10 studies are biased. C, Seven out of 10 studies are biased, but unbiased studies comprise the largest subgroup of studies that reported the same result. D, Seven out of 10 studies are biased, and biased studies comprise the largest subgroup of studies that reported the same result

Figure 2 illustrates the assumptions underlying the combined mean, median and mode in a hypothetical meta‐analysis of 10 studies, sorted in ascending order of their *β*_*j*_s. The true effect *β* is zero. For simplicity, all studies have the same weight and no sources of heterogeneity other than bias are present. Chiefly:

- when all 10 studies (ie, 100%) are unbiased (Panel A), all three estimators identify the true effect (zero);
- when 4 out of 10 studies are biased (Panel B), the mean is biased, but the median and the mode are unbiased;
- when 7 out of 10 studies are biased (Panel C), or whenever more than 50% of studies are biased in general, and ZEMBA is satisfied, both the mean and the median are biased, but not the mode; and
- when more than 50% of the studies are biased (Panel D) and ZEMBA is violated, all estimators are biased.

An attractive property of the weighted median and MBE is that they are naturally robust central tendency statistics, but do not make any specific assumptions about the bias mechanism at play. Therefore, they are robust to a range of possible causes of small‐study effects. However, as Figure 2 illustrates, these estimators are not guaranteed to provide consistent estimates of *β*, failing to do so when their identifying assumptions are violated. Nevertheless, the assumptions they require are weaker than those of the standard weighted mean.

### 3.5 Regression‐based extrapolation and trim‐and‐fill

We compared the weighted median and the MBE with two meta‐analysis estimators developed to adjust for small‐study effects. The first, described by Moreno et al,^11^ is extrapolation to the estimated effect of the intervention in a study of infinite size based on a linear regression weighted by $\sigma_{j}^{-2}$. This estimator assumes a linear relationship between the *b*_*j*_s and *σ*_*j*_s so that *b*_*j*_ = *β*_1_*σ*_*j*_. Plugging this expression for *b*_*j*_ in Equation (1) to the regression‐based extrapolation model yields:

(6) $${\hat{\beta }}_{j}=\beta_{0}+\beta_{1}\sigma_{j}+\sigma_{j}\varepsilon_{j}.$$

In Equation (6), *β*_0_ is the estimated effect in a study of infinite size, obtained by extrapolation based on the model assumptions. *β*_1_ is the parameter that allows accounting for bias via nonzero *b*_*j*_s so that testing *H*_0_ : *β*_1_ = 0 is a test for the presence of small‐study effects. Indeed, this test has been shown^5^ to be identical to the test of funnel plot asymmetry proposed by Egger et al.^4^ For simplicity, Equation (6) shows the fixed‐effect regression‐based extrapolation model, which can be extended into an additive or multiplicative random effects model^23^; the latter was used in the simulations and real data examples described below. This approach is illustrated in Supplementary Figure 1 (panels A and C).

The second estimator is trim‐and‐fill, a nonparametric data augmentation method that estimates the number of missing studies (eg, due to publication bias) by suppressing (or “trimming”) the most extreme studies from one side of the funnel plot. Then, the data are augmented so that the funnel plot is more symmetric. The augmented data are then used to calculate the combined effect.^10^ In our simulations and real data examples, we used a random effects model throughout the trim‐and‐fill process (sometimes referred to as random‐random effects trim‐and‐fill) and the *L*0 estimator to estimate the number of missing studies. This approach is illustrated in Supplementary Figure 1 (panels B and D).

## 4 REANALYSIS OF PUBLISHED META‐ANALYSES

To illustrate the application of the proposed meta‐analysis estimators and compare them with existing approaches, we reanalyzed the four meta‐analysis datasets described in Section 2 (Table 1).

**Table 1:** Combined estimates with 95% confidence intervals for different meta‐analysis datasets and estimators

Estimator	Dataset			
	Catheter^a^	Aspirin^a^	Sodium^b^	Streptokinase^c^
	*I*^2^ = 60%	*I*^2^ = 10%	*I*^2^ = 99%^d^	*I*^2^ = 34%
	*γ* = − 3.05 (*P* = 0.007)	*γ* = 2.11 (*P* = 5.2 × 10^−9^)	*γ* = 2.60 (*P* = 0.679)^d^	*γ* = − 0.06 (*P* = 0.868)
Weighted mean	0.47 (0.38; 0.58)	3.43 (2.96; 3.98)	1.48 (1.39; 1.57)	0.82 (0.76; 0.88)
Regression‐based extrapolation	1.27 (0.70; 2.31)	1.03 (0.71; 1.48)	1.24 (−0.72; 3.21)	0.83 (0.72; 0.94)
Trim‐and‐fill	0.45 (0.31; 0.65)	2.87 (2.38; 3.47)	2.62 (0.99; 4.26)	0.81 (0.71; 0.93)
Weighted median	0.57 (0.43; 0.75)	2.99 (2.41; 3.72)	0.62 (0.52; 0.72)	0.83 (0.75; 0.91)
Weighted mode	0.57 (0.44; 0.75)	2.55 (1.82; 3.56)	0.61 (0.51; 0.70)	0.83 (0.75; 0.90)

a Odds ratio.

b Mean difference.

c Risk ratio.

d Removing outlying studies substantially attenuates both *γ* and *I*^2^ (see Section 4).

*Notes*: *I*^2^: between‐study inconsistency. *γ*: Egger test's coefficient (ie, slope in inverse variance weighted linear regression of effect estimates on SEs).

In our reanalysis of the catheter dataset (for which both *γ* and *I*^2^ were high), the weighted mean yielded an odds ratio of bloodstream infection of 0.47 (95% CI: 0.38; 0.58), while the weighted median and the MBE yielded the same smaller (in magnitude) estimate of 0.57 (95% CI: 0.44; 0.75). Trim‐and‐fill yielded 0.45 (95% CI: 0.31; 0.65), similar to the weighted mean results. Regression‐based extrapolation yielded a qualitatively different estimate of 1.27 (95% CI: 0.70; 2.31). This is likely an over‐correction, especially given that the individual‐study odds ratio estimates in the data ranged from 0.09 to 0.83 (Supplementary Figure 1B).

For the aspirin dataset (which presented low *I*^2^ and marked asymmetry), the combined odds ratio estimates of at least 50% of pain relief comparing active treatment to placebo were 3.43 (95% CI: 2.96; 3.98) for the weighted mean, 2.99 (95% CI: 2.41; 3.73) for the weighted median, and 2.55 (95% CI: 1.78; 3.63) for the MBE. Trim‐and‐fill yielded an odds ratio of 2.87 (95% CI: 2.38; 3.47), which was closer to the weighted median and the MBE results than to the weighted mean. Regression‐based extrapolation yielded an odds ratio of 1.03 (95% CI: 0.71; 1.48), suggesting no effect of aspirin whatsoever (and again likely over‐corrected—Supplementary Figure 1D).

For the sodium dataset, removing the outlying study (as classified by Leyvraz et al^19^) with the largest weight reduced between‐study inconsistency (*I*^2^ = 87%). Removing both studies eliminates between‐study inconsistency (*I*^2^ = 0%). Removing these studies also substantially attenuates asymmetry (*γ*= − 0.22 and *γ* = 0.68, respectively). This suggests that, unlike the previous examples, between‐study inconsistency and asymmetry mostly stemmed from two studies (out of 13). Without removing any studies, the weighted mean, regression‐based extrapolation, and trim‐and‐fill estimators suggested an average decrease in systolic blood pressure due to sodium intake‐lowering interventions of 1.48 (95% CI: 1.39; 1.57), 1.24 (95% CI: −0.72; 3.21), and 2.62 (95% CI: 0.99; 4.26) mmHg, respectively. All these results are higher than the bulk of the funnel plot (Figure 1C). Conversely, the weighted median and the MBE yielded combined estimates of 0.62 (95% CI: 0.52; 0.72) and 0.61 (95% CI: 0.51; 0.70), respectively, which is in line with the majority of the studies and located within the bulk of the plot. Indeed, these results are similar to those obtained by Leyvraz et al^19^ after they explicitly excluded these two studies from the meta‐analysis.

For the streptokinase dataset (which was used as a positive control), the combined risk ratio estimate comparing treatment and control groups was 0.82 (95% CI: 0.76; 0.88) using the weighted mean. Results from the other four estimators ranged from 0.81 to 0.83. Given that the largest trial^24^ corresponded to a substantial proportion of the total weight in the meta‐analysis, the observed consistency between the estimators could simply be that they were all driven by this large study. However, in a sensitivity analysis where this study was removed, there was no material effect on the results. Therefore, the observed consistency between the approaches in this example was likely due to the symmetry of the data rather than to the influence of a single large trial.

The results above indicate that the weighted median and the MBE are less influenced by outlying studies compared to the weighted mean, regression‐based extrapolation, and trim‐and‐fill. This is a useful property at least for sensitivity analysis purposes, especially for meta‐analyses with a small number of datapoints (and thus more sensitive to outliers). The proposed estimators appeared more robust to the presence of small‐study effects than the weighted mean. In a dataset with substantial asymmetry but low between‐study inconsistency (the aspirin dataset), the weighted median and the MBE gave similar results to the trim‐and‐fill estimator. In a dataset with substantial asymmetry and between‐study inconsistency (the catheter dataset), the proposed estimators were less influenced by the left skew in the funnel plot than the trim‐and‐fill, which gave very similar results to the weighted mean. This suggests that presence of between‐study inconsistency has a more limited effect on the robustness of the proposed estimators to small‐study effects than on trim‐and‐fill (in the simulations, these estimators are compared in scenarios with varying degrees of between‐study inconsistency). In the datasets with asymmetry, regression‐based extrapolation yielded results that were likely overcorrected.

## 5 SIMULATION STUDY

### 5.1 Brief description

We performed a simulation study to evaluate the performance of the weighted mean, regression‐based extrapolation, trim‐and‐fill, weighted median and MBE. Summary data were generated using Equation (1). We assume that each study measured a binary exposure variable *X*~Bernoulli(0.5) (eg, an intervention: yes = 1, no = 0) and a continuous outcome variable *Y* with variance equal to one. Therefore, the standard error of the mean difference is one for all values of *j*, and $\sigma_{j}=\sqrt{4/n_{j}}$. We assume that studies range in size from *n*_1_ to *n*_2_ uniformly, so that *n*_*j*_~Uniform(*n*_1_, *n*_2_).

Data were generated to contain two forms of bias (see Box 1 for their general principles). Type (a) bias is a fundamental property of each study (eg, bias due to lack of intervention allocation concealment, or residual confounding in the case of meta‐analyses of observational studies). For simulations under type (a) bias, the proportion of biased studies is dictated by the parameter *δ* ∈ [0, 1]. Among biased studies, *b*_*j*_ varies linearly with *n*_*j*_.

Type (b) bias is the result of publication bias, not a property of each study. For simulations under type (b) bias, we assumed (in common with most publication bias models) that results achieving conventional levels of statistical significance are more likely to be published. Therefore, *l*_*j*_ was defined to correspond to the maximum one‐sided P‐value (null hypothesis: true mean difference ≤ 0) allowed for publication for a given study size (*p*_*j*_), up to *N*. That is, for *n*_*j*_ ≥ *N*, then there are no *P*‐value requirements for publication, that is, *p*_*j*_ = 1 for all values of *j* (because the study size is sufficient for publication regardless of its results). For *n*_*j*_ < *N*, larger studies are more likely to be published than smaller studies, where *p*_*j*_ is a nondecreasing function of *n*_*j*_. Therefore, *N* is the minimal study size required for the *P*‐value to have no influence on the publication probability, which can be used to increase (if *N* is larger) or decrease (if *N* is smaller) the degree of type (b) bias. We evaluated four distinct functions: linear, square root, quadratic, and step function. The relationship between *p*_*j*_ and *n*_*j*_ in each one of these four type (b) bias mechanisms is illustrated in Supplementary Figure 2.

In all simulations, *K* was set to 5, 10, 30, or 50. In Scenarios 1‐6, *β* = 0. In Scenario 1, there is neither type (a) nor type (b) bias. Scenario 2 evaluated type (a), but not type (b), bias. Scenarios 3‐6 evaluated type (b) bias (but not type (a) bias), where *p*_*j*_ was a linear, square root, quadratic, or step function (respectively) of *n*_*j*_, up to *N*. Scenario 7 was identical to Scenario 1, except that *β* = 0.02. Table 2 describes the main characteristics and aims of each scenario.

**Table 2:** Brief description of the simulation scenarios

Scenario	*β* a	Bias type	Aim
1	0	None	Assess bias and FRR^b^ under neither treatment effect nor small‐study effects, for various numbers of studies included in the meta‐analysis.
2	0	Type (a)	Assess bias and FRR^b^ in the presence of type (a) bias, for various numbers of studies included in the meta‐analysis.
3	0	Type (b)	Asses bias and FRR^b^ in the presence of type (b) bias, for various numbers of studies included in the meta‐analysis. In scenario 3, the minimum P‐value required for publication is a linear function of study size.
4	0	Type (b)	Same as scenario 3, except that the minimum P‐value required for publication is a square root function of study size.
5	0	Type (b)	Same as scenario 3, except that the minimum P‐value required for publication is a quadratic function of study size.
6	0	Type (b)	Same as scenario 3, except that the minimum P‐value required for publication is a step function of study size.
7	0.02	None	Assess power to detect a non‐zero treatment effect in the absence of small‐study effects, for various numbers of studies included in the meta‐analysis.

Abbreviation: FRR, false‐rejection rate.

a Treatment effect.

b Since *β* = 0 in scenarios 1‐6, the FRR under small‐study effects is simply the overall rejection rate.

**Figure 3:**
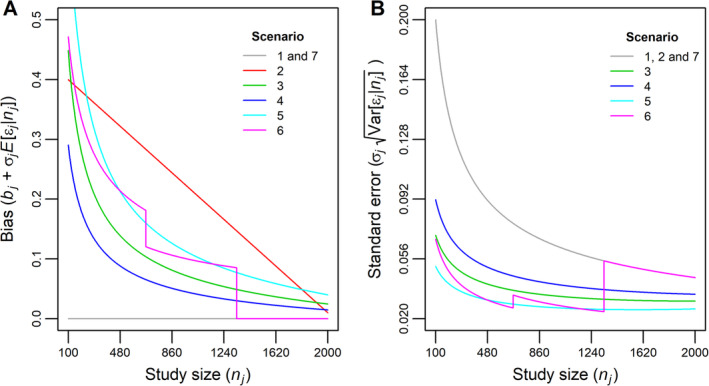
Illustration of the relationship between bias and *n*_*j*_ (panel A), and between standard error and *n*_*j*_ (panel B), induced by different models of small‐study effects [Colour figure can be viewed at wileyonlinelibrary.com]

The functional relationship between the bias (ie, $E[{\hat{\beta }}_{j}\left|n_{j}\right]-\beta =b_{j}+\sigma_{j}E\left[\varepsilon_{j}|n_{j}\right]$) and *n*_*j*_, and between standard error (ie, $\sigma_{j}\sqrt{\mathrm{Var}\left[\varepsilon_{j}|n_{j}\right]}$) and *n*_*j*_, in scenarios 1‐7 is illustrated in Figure 3.

The data generating mechanism and simulation parameters are described in more detail in the Supplement. Mean combined effect estimates, standard errors, coverage, and rejection rates of 95% confidence intervals were computed for the weighted mean, weighted median, MBE, regression‐based extrapolation, and trim‐and‐fill estimators across 10 000 simulated datasets. All analyses were performed using R.^25^ We used the “metafor” package to calculate the *I*^2^ statistic and to perform the weighted mean and trim‐and‐fill estimators.^26^ The “truncnorm” package was used to generate random draws from the standard truncated normal distribution.^27^ The “doParallel” package was used for parallel computing.^28^

### 5.2 Simulation study results

Simulation scenario 1 showed that confidence intervals for the weighted mean, weighted median, and MBE are valid under the null in the sense that they all achieve at least 95% coverage when *β* = 0 and in absence of small‐study effects, although only the weighted mean had exact 95% coverage (Supplementary Figure 3 and Supplementary Table 1). Regression‐based extrapolation showed under‐coverage when the number of studies was small, but this attenuated as the number of studies increased. Conversely, trim‐and‐fill showed under‐coverage that increased with number of studies, indicating that its confidence intervals are invalid (at least in our implementation of the estimator). The weighted mean had the smallest standard errors, followed by trim‐and‐fill, which was slightly more precise than the weighted median. The MBE was less precise than the latter, but substantially more precise than regression‐based extrapolation.

**Figure 4:**
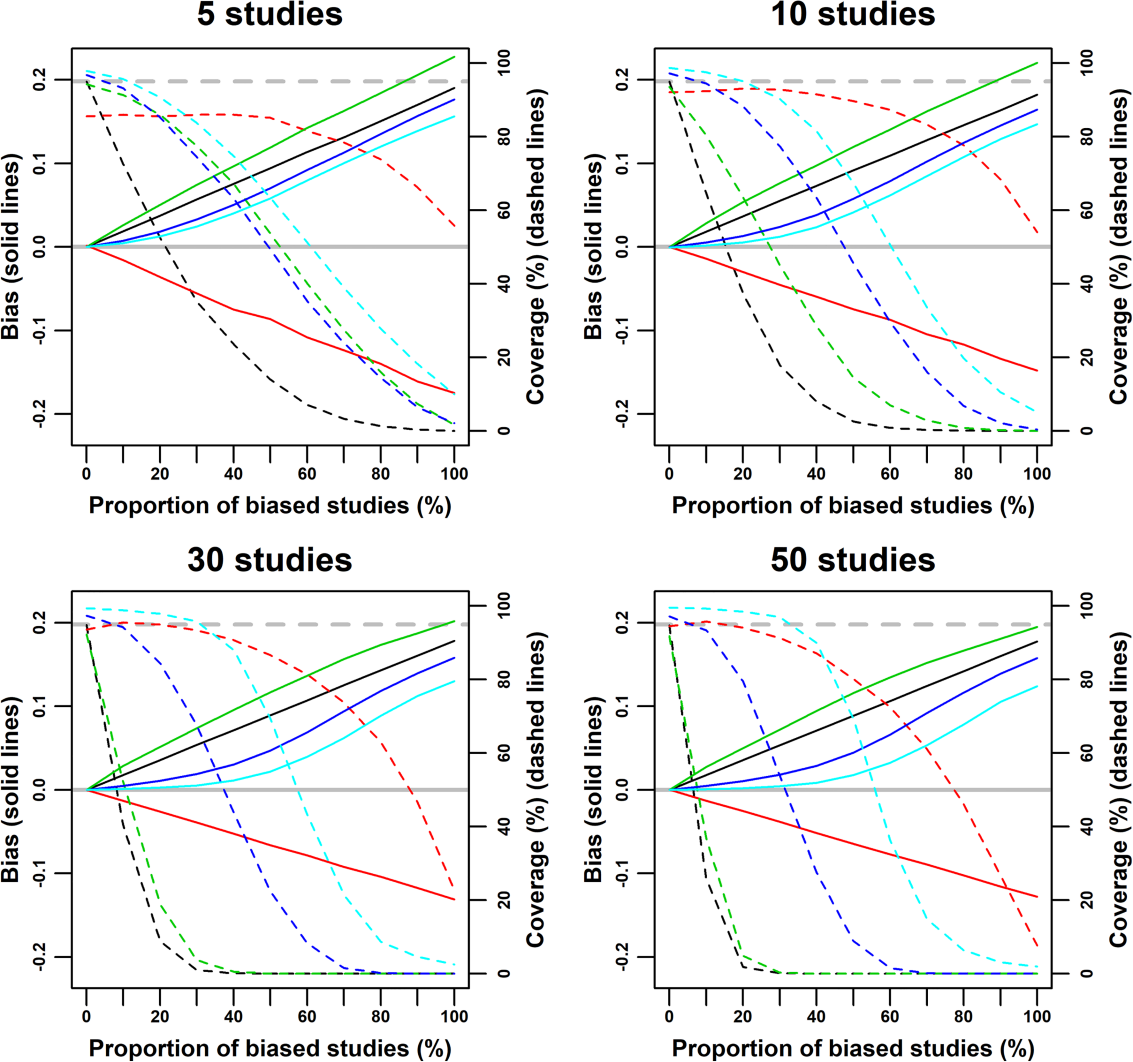
Bias (solid lines) and coverage (dashed lines) of the weighted mean (black), regression‐based extrapolation (red), trim‐and‐fill (green), weighted median (dark blue), and mode‐based estimate (light blue) under scenario 2: zero true effect (ie, *β* = 0), small‐study effects through the bias term *b*_*j*_, and study sizes uniformly ranging from 100 to 5000 individuals. The grey line indicates zero bias. The dashed grey line indicates 95% coverage [Colour figure can be viewed at wileyonlinelibrary.com]

Supplementary Table 2 shows that scenario 2 leads to high values of *I*^2^ and *γ*. Under this scenario, the weighted median was less biased than the weighted mean, and the MBE was the least biased among all approaches (Figure 4). Those differences became more apparent as the number of studies increased. Trim‐and‐fill was more biased than the standard weighted mean, and regression‐based extrapolation substantially overcorrected for the bias.

**Figure 5:**
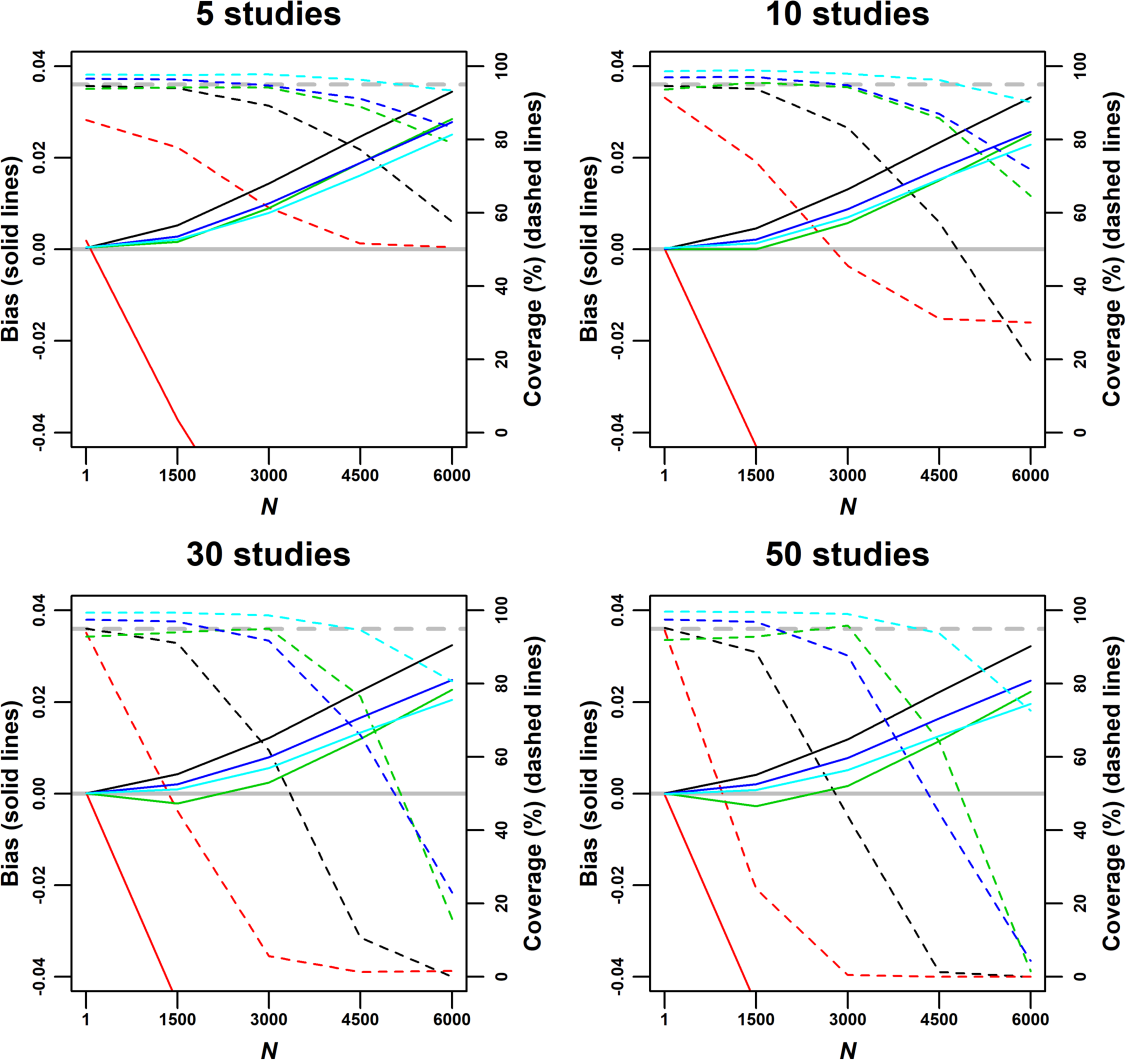
Bias (solid lines) and coverage (dashed lines) of the weighted mean (black), regression‐based extrapolation (red), trim‐and‐fill (green), weighted median (dark blue), and mode‐based estimate (light blue) under scenario 3: zero true effect (ie, *β* = 0), small‐study effects through publication bias (assuming a linear relationship between *p*_*j*_ and *n*_*j*_), and study sizes uniformly ranging from 100 to 5000 individuals. *p*_*j*_: maximum P‐value allowed for publication for a study with *n*_*j*_ participants. *N*: study size threshold, with studies larger than or equally sized to *N* not being affected by small‐study effects. The grey line indicates zero bias. The dashed grey line indicates 95% coverage [Colour figure can be viewed at wileyonlinelibrary.com]

Scenario 3 leads to high asymmetry, but not a substantial inflation of *I*^2^ (Supplementary Table 3), and the bias in the combined estimates was much smaller than in scenario 2. Again, regression‐based extrapolation substantially overcorrected for small‐study effects, and the weighted median and MBE were less biased than the weighted mean (Figure 5). However, the performance of trim‐and‐fill relative to the weighted median and the MBE was substantially different than in scenario 2: if the number of studies is low (*K* = 5), trim‐and‐fill performed similarly to the weighted median, but was more biased than the MBE; for *K* = 10, it outperformed the weighted median and performed similarly to the MBE; for larger values of *K*, trim‐and‐fill was generally less biased than the other estimators, unless all studies were affected by small‐study effects (in this case, *N* = 6000). However, as the number of studies increased, trim‐and‐fill overcorrected for small‐study effects when *N* = 1500. In general, the differences between the weighted median, the MBE and trim‐and‐fill were much less marked in scenario 3 than in scenario 2; indeed, the coverage of the weighted median and trim‐and‐fill was similar for all values of *K*.

In scenario 4, small‐study effects resulted in less marked asymmetry than for scenario 3 and in reduced *I*^2^, that is, under‐dispersion (Supplementary Table 4). In general, results were similar to scenario 3 (see Supplementary Figure 4), with two main differences. First, the weighted median had better coverage than trim‐and‐fill, unless *K* = 50 and *N* = 4500. Second, the overcorrection showed by trim‐and‐fill in scenario 3 was more apparent, especially for larger values of *K*. Scenario 5 was in between scenarios 2 and 3 regarding *γ* and *I*^2^ (Supplementary Table 5). In this scenario, trim‐and‐fill was more biased than the weighted median and the MBE when the number of studies was low (*K* = 5 or *K* = 10), and in between them when there were more studies (*K* = 30 or *K* = 50). The difference between the weighted median and the MBE was small regardless of the number of studies (Supplementary Figure 5). In scenario 6, there was more between‐study inconsistency compared with the last scenario, but less than in scenario 2 (Supplementary Table 6). The weighted median and the MBE performed substantially better than the other estimators (as shown in Supplementary Figure 6), with the MBE being close to unbiased in all cases when the number of studies was large (*K* = 30 or *K* = 50).

Supplementary Table 7 and Supplementary Figure 7 display the performance of the estimators in detecting an effect in the absence of small‐study effects (scenario 7). The weighted mean was the estimator with the highest power to detect a nonzero treatment effect, followed by trim‐and‐fill and the weighted median. Importantly, trim‐and‐fill was slightly more precise than the weighted median, but had substantially more power due to its under‐coverage (which increased with number of studies and study size). The MBE was substantially more precise than regression‐based extrapolation, but had lower power due to under‐coverage of the latter when the number of studies was low.

Our simulation study corroborated the well‐known notion that the weighted mean is biased in the presence of small‐study effects (either type (a) and type (b)). In all small‐study effects mechanisms, regression‐based extrapolation overcorrected the treatment effect (this is discussed in more detail in the Supplementary Text). Trim‐and‐fill was more biased in the presence of type (a) bias (which lead to substantial between‐study inconsistency) than the weighted mean. Trim‐and‐fill was less affected by type (b) than type (a) bias, thus highlighting the dependence of this estimator to the underlying data generating mechanism. Moreover, for most variations of type (b) bias, this estimator presented more bias than the weighted median and the MBE, as well as under‐coverage in the absence of any small‐study effects. Conversely, the weighted median and the MBE had confidence intervals with coverage ≥95% in the absence of small‐study effects and were relatively robust to both type (a) and type (b) bias.

## 6 DISCUSSION

We have proposed the weighted median and the MBE for meta‐analysis as approaches that are robust to the presence of small‐study effects and outliers. Application to a series of examples indicates that both approaches give sensible estimates of the intervention effect in real meta‐analyses where small‐study effects are suspected, even when regression‐based extrapolation or trim‐and‐fill do not. They also give similar results to the weighted mean and other meta‐analysis approaches in absence of funnel plot asymmetry. Our real data examples also illustrated the robustness of the proposed estimators to outliers. Our comprehensive simulation study confirmed these findings, and showed that these estimators are less influenced by small‐study effects than the conventional weighted mean and previously proposed approaches to estimate intervention effects in the presence of small‐study effects. Software for their implementation is provided in the Supplementary Material.

There are several strategies to investigate the presence and degree of small‐study effects in meta‐analysis, all of which have limitations.^6^, ^29^ If, after careful examination, small‐study effects are suspected, we recommend that investigators apply the weighted median and the MBE in addition to standard estimators as sensitivity analyses. These estimators reduce the influence of small and/or outlying studies without excluding them formally from the meta‐analysis. Exclusion often involves arbitrary study size cut‐offs and artificially reduces the heterogeneity in the data.

When applying the weighted median and the MBE, it is important not to rely entirely on “statistical significance”, especially given that they are less precise than the weighted mean. Instead, meta‐analysis authors should examine confidence intervals for the different estimators and assess their consistency with standard meta‐analysis estimates. In general, the weighted median and the MBE will be robust when studies that provide consistent effect estimates receive most of the weight in the fixed‐effect meta‐analysis. This might occur in a meta‐analysis with just one or two large studies providing consistent estimates, despite the inclusion of many other biased, smaller studies. Conversely, the weighted median and the MBE will give misleading results when the majority of the weight in the analysis stems from biased studies and, in the case of the MBE, the magnitude of the individual study biases are very similar (as illustrated in Figure 2D). The Cochrane tool for assessing risk of bias in randomized trials^30^ could be used as a guide to the likely proportion of biased studies in a given meta‐analysis, and to the value of applying these techniques. As such the proposed estimators are a natural extension of exploring between study heterogeneity due to perceived risk of bias.^30^

We have presented the weighted median and the MBE assuming treatment effect homogeneity. Under this assumption, any heterogeneity between effect estimates is indicative of bias. By supplementing this assumption with additional assumptions (such as the 50% rule for the weighted median or ZEMBA for the MBE) then allows consistent estimation of the treatment effect even in the presence of (some forms of) small‐study effects. In the absence of bias and in the presence of heterogeneous treatment effects, the weighted mean, weighted median, and MBE estimate the inverse‐variance weighted average, median, and modal treatment effects (in the case of unique treatment effects for each study, the latter would simply be the most precise effect estimate). However, in this case, a more sensible approach would be to perform a random effects meta‐analysis to estimate the average treatment effect. But doing so requires the assumption that there is no form of bias in any of the studies included in the meta‐analysis (because all studies have non‐zero weight in the meta‐analysis), which may itself not be warranted at least in some applications. This illustrates the more general notion that relaxing one assumption often requires more contrived versions of one or more other assumptions. In this case, assuming absence of bias allows interpreting systematic differences between studies as being solely due to treatment effect heterogeneity (and thus a random effects weighted mean can be used to estimate the average treatment effect), while assuming treatment effect homogeneity allows interpreting such differences as being solely due to bias (and thus estimators such as the weighted median and the MBE can be used to estimate the treatment effect under some forms of bias).

Importantly, the estimators proposed here cannot be regarded as providing a general “correction” for funnel plot asymmetry or heterogeneity between studies. Heterogeneity between studies should be expected in real meta‐analyses,^31^ and exploring whether it is explained by measured study characteristics (eg, via subgroup analyses and meta‐regression) may yield important insights regarding treatment effect modification and/or potential sources of bias. Such insights cannot be achieved by simply applying the proposed estimators, nor any other approach that yields a single point estimate. This is especially relevant for the MBE estimator, which assumes that there is a subset of homogeneous studies that yield consistent estimates of the treatment effect. Therefore, ideally the proposed estimators would be applied if plausible effect modifiers do not account for observed heterogeneity between studies, or if there is residual heterogeneity within subgroups (although in this case the number of studies per subgroup may be prohibitive for meaningful comparisons between different estimators). Otherwise, the MBE can be used as a sensitivity analysis and interpreted as a test of the sharp null hypothesis (ie, the hypothesis that the intervention has no effect whatsoever on anyone in the population). Supplementation with further assumptions would allow some learning about the average treatment effect. For example, if the true treatment effect can be assumed to be monotonic (ie, in the same direction for all studies), then the MBE can be interpreted as a test of the direction of the treatment effect.

As mentioned in Section 3.3.3, the MBE is just one way of exploiting the ZEMBA assumption to mitigate the influence of small‐study effects in meta‐analysis. There are many other ways of estimating the mode of continuous data, such as the half‐sample mode method,^21^ Grenander's estimators,^32^ model‐averaging,^33^ and explicit selection^34^ methods. Even restricting to only kernel‐based methods such as the MBE, there are many available choices of bandwidths and kernels. It is therefore possible that there are estimators more adequate than the MBE to exploit the ZEMBA assumption in meta‐analysis, a topic that remains to be investigated. The goal of the present study was to present ZEMBA as an alternative identification assumption, and compare the performance of one estimator that relies on this assumption (the MBE) against established meta‐analysis estimators.

In summary, many systematic reviews and meta‐analyses contain studies that are methodologically flawed and likely biased.^35^ We have proposed new weighted median and mode‐based estimators that provide inferences that are robust to small‐study effects under a variety of reasonable simulation scenarios. Their application in real datasets supports their likely utility as a sensitivity analysis in comparison to standard mean‐based meta‐analytic estimates. We hope that these estimators will be used to strengthen the conclusions of systematic reviews and meta‐analyses.

## CONFLICT OF INTEREST

The authors declare no conflicts of interest.

## Supporting information

Data S1. Supplementary Material

## Data Availability

Data sharing is not applicable to this article as no new data were created or analysed in this study.
