# Supplementary material for: The median and the mode as robust meta‐analysis estimators in the presence of small‐study effects and outliers
Source: Res Synth Methods. 2020 Mar 10;11(3):397–412. doi: 10.1002/jrsm.1402 (PMC7359861; doi:10.1002/jrsm.1402)
Supplement: Supplementary file 1 — Data S1. Supplementary Material [file JRSM-11-397-s001.docx]

**Supplementary Material**

Summary

[Supplementary Text 2](#_Toc535585686)

[1. Simulation study 2](#_Toc535585687)

[2. Bias in regression-based extrapolation due to small study effects 4](#_Toc535585688)

[Supplementary Tables 6](#_Toc535585689)

[Supplementary Figures 13](#_Toc535585690)

[Software code (R language) 21](#_Toc535585691)

[Weighted median 21](#_Toc535585692)

[Mode-based estimate 23](#_Toc535585693)

**Supplementary Text**

## 1. Simulation study

In section 4.1, we provided a simpler and more accessible description of the simulation study. Here we provide a more complete and technical description.

Summary data were generated using equation (1). We assume that each study measured a binary exposure variable $X\sim Bernoulli(0.5)$ (e.g., an intervention: yes=1, no=0) and a continuous outcome variable $Y$ with variance equal to one. Therefore, the standard error of the mean difference is one for all values of $j$, and $\sigma_{j}=\sqrt{4/{n_{j}}}$. We assume that studies range in size from $n_{1}$ to $n_{2}$ uniformly, so that $n_{j}\sim\mathrm{Uniform}\left( n_{1},n_{2} \right).$ Two forms of bias – type (a) and type (b) were simulated (see Box 1 for details).

**1.1. Type (a) bias**

The value of the bias term $b_{j}$ was defined as the following linear function of study size: $b_{j}={I_{b}}_{j}\left( 0.5-{1\times10}^{-4}\left( n_{j}-100 \right) \right)$. From this, if $n_{j}=100$ (the smallest study size in our simulations), then $b_{j}=0.5{I_{b}}_{j}$. If $n_{j}=5000$ (the largest study size in our simulations), then $b_{j}=0.01{I_{b}}_{j}$.

The indicator ${I_{b}}_{j}\sim\mathrm{Bernoulli}(\delta)$, with $\delta\in[0,1]$, dictates the presence (${I_{b}}_{j}=1$) or absence (${I_{b}}_{j}=0$) of bias. Therefore, the expected number of studies suffering from bias equals $\delta K$.

**1.2. Type (b) bias**

This bias was generated through $\varepsilon_{j}$ by varying $l_{j}$ according to study size. Typically, publication bias mechanisms assume that results that achieve conventional levels of statistical significance are more likely to be published. Therefore, in our simulations, $l_{j}$ was defined to correspond to the maximum one-sided P-value (null hypothesis: true mean difference ≤0) allowed for publication for a given study size ($p_{j}$). That is:

$$l_{j}=Q\left( 1-p_{j} \right)\text{ (S1)},$$

where $Q(p)$ is the quantile function for the Student’s t distribution with $n_{j}-1$ degrees of freedom. For example, if $p_{j}=0.025$ and $n_{j}=1000$, then $l_{j}=Q\left( 1-p_{j} \right)= Q\left( 0.975 \right)\approx1.96$. This situation can be interpreted as studies with 1000 participants only being publishable if the reported one-sided P-value is $\leq0.025$.

We generated publication bias by defining $p_{j}$ as various functions of $n_{j}$, as described below.

i) $p_{j}$ as a continuous function of $n_{j}$ (up to $N$).

Here, $p_{j}=\min\left( f\left( \frac{n_{j}}{N} \right),1 \right)$, where $f\left( \frac{n_{j}}{N} \right)$ is some non-decreasing function of $\frac{n_{j}}{N}$ and $N$ is some upper threshold study size threshold at which $p_{j}=1$ (and therefore $l_{j}=-\infty$) for all $n_{j}\geq N$. We compared three distinct functions:

- Identity: $f\left( \frac{n_{j}}{N} \right)=\frac{n_{j}}{N}$;
- Square root: $f\left( \frac{n_{j}}{N} \right)=\sqrt{\frac{n_{j}}{N}}$; and
- Quadratic: $f\left( \frac{n_{j}}{N} \right)=\left( \frac{n_{j}}{N} \right)^{2}$.

ii) $p_{j}$ as a step function of $n_{j}$.

Here, $p_{j}$ is defined as a piecewise function of $n_{j}$ classifying studies into small, medium or large. That is:

$$p_{j}=\left\{ \begin{matrix} p_{small} & \mathrm{if}n_{j}\leq N_{small} \\ p_{medium} & \mathrm{if}N_{small}<n_{j}<N_{large} \\ p_{large} & \mathrm{if}N_{large}\leq n_{j} \end{matrix} \right.\text{ (}\text{S2}\text{)},$$

where $1\leq N_{small}\leq N_{large}$ and $0\leq p_{small}\leq p_{medium}\leq p_{large}\leq1$. Therefore, studies classified in the same group have the same P-value requirements for publication, and the relationship between $p_{j}$ and $n_{j}$ follows a step function.

**2.3. Simulation scenarios**

We evaluated seven simulation scenarios. In all cases, $K$ was set to 5, 10, 30 or 50. In Scenarios 1-6, $\beta=0$. Further details are given below.

- Scenario 1: No small study effects. The data generating mechanism (1) simplifies to $\hat{\beta}_{j}=\sigma_{j}\varepsilon_{j}$, where $\varepsilon_{j}\sim N(0,1,-\infty,\infty)$. Study size chosen as: i) $n_{1}=100$, $n_{2}=1000$; and ii) $n_{1}=1000$, $n_{2}=5000$.
- Scenario 2: Type (a) bias only: the data generating mechanism in (1) equals $\hat{\beta}_{j}=b_{j}+\sigma_{j}\varepsilon_{j}$, for $\varepsilon_{j}\sim N(0,1,-\infty,\infty)$. Study sizes ranged between $n_{1}=100$ and $n_{2}=5000$ (these values were also used in scenarios 3-6), and the proportion of biased studies $\delta$ was varied between 0 and 1 in steps of 0.1.
- Scenarios 3-5: Type (b) bias only, yielding the data generating mechanism $\hat{\beta}_{j}=\sigma_{j}\varepsilon_{j}$, $\varepsilon_{j}|n_{j}\sim N(0,1,l_{j},\infty)$, and $l_{j}=Q\left( 1-\min\left( f\left( \frac{n_{j}}{N} \right),1 \right) \right)$ We assumed a linear (scenario 3), square root (scenario 4) or quadratic (scenario 5) relationship between $p_{j}$ and $n_{j}$. $N$ was set to 1 (i.e., no small study effects), 1500, 3000, 4500 and 6000.
- Scenario 6: Type (b) bias only, assuming a step-function relationship between $p_{j}$ and $n_{j}$. This used the same mechanism as in Scenarios 3-5 except with $p_{j}$ defined following equation (S2) and where $p_{small}=0.025$, $p_{medium}=0.15$ and $p_{large}=1$ (the latter implying that large studies have no P-value requirements for publication) were kept constant. Cut-offs to classify studies into small, medium or large varied as follows: i) $N_{small}=N_{large}=1$ (i.e., no small study effects); ii) $N_{small}=500$, and $N_{large}=1000$; iii) $N_{small}=1000$, and $N_{large}=2000$; iii) $N_{small}=2000$, and $N_{large}=4000$.
- Scenario 7: identical to Scenario 1, except that $\beta=0.02$.

## 2. Bias in regression-based extrapolation due to small study effects

In the catheter and aspirin meta-analyses (both of which presented evidence of funnel plot asymmetry), the combined effect estimate from regression-based extrapolation ($\hat{\beta}_{RBE}$) was outside the range of individual study estimates (the $\hat{\beta}_{j}$’s, following the notation in equation (1)). More specifically, the catheter dataset presented a positive correlation between the $\hat{\beta}_{j}$’s and $\sigma_{j}^{-1}$’s ($r$=0.73), and its regression-based extrapolation combined ln(odds ratio) estimate $\hat{\beta}_{RBE}$=0.24 was largest than $max(\hat{\beta}_{j})$=-0.19. The aspirin dataset presented a negative correlation between the $\hat{\beta}_{j}$’s and $\sigma_{j}^{-1}$’s ($r$=-0.69), and its regression-based extrapolation combined ln(odds ratio) estimate $\hat{\beta}_{RBE}$=0.03 was smaller than $min(\hat{\beta}_{j})$=0.14. These results suggest, but do not prove, that regression-based extrapolation was overcorrecting for small study effects. This was corroborated in our simulations, where regression-based extrapolation yielded negative combined estimates in the presence of positive bias and no treatment effect.

As described in the main text (section 3.6), regression-based extrapolation explicitly assumes that there is a linear relationship between the $b_{j}$’s and $\sigma_{j}$’s. Therefore, it is prone to bias if the data generating mechanism leads to a non-linear relationship between the $b_{j}$’s and $\sigma_{j}$’s (or, more generally, if the regression model is miss-specified).

In our simulations bias can be defined as $b_{j}+{\sigma_{j}E[\varepsilon}_{j}|n_{j}]$, and the standard error as $\sigma_{j}\sqrt{\mathrm{Var}\left[ \varepsilon_{j}{|n}_{j} \right]}$. Therefore, the regression-based extrapolation model will only be correctly specified if $b_{j}+{\sigma_{j}E[\varepsilon}_{j}\left| n_{j} \right]=\beta_{0}\sigma_{j}\sqrt{\mathrm{Var}\left[ \varepsilon_{j}{|n}_{j} \right]}$; in other words, if $b_{j}+{\sigma_{j}E[\varepsilon}_{j}\left| n_{j} \right]$ and $\sigma_{j}\sqrt{\mathrm{Var}\left[ \varepsilon_{j}{|n}_{j} \right]}$ are linearly related. However, Supplementary Figure 8 shows that in all our small study effects mechanisms the relationship between bias and standard error is non-linear, thus leading to bias in $\beta_{RBE}$.

The small study effects mechanisms evaluated in our simulations represent plausible data-generating mechanisms that include the main features of typical mechanisms of small study effects. Particularly, in the case of publication bias, it is likely that selection is not influenced by precision itself, but by sample size (with larger studies being more likely to be published than smaller studies) and statistical significance levels (with studies that achieve conventional levels of statistical significance being more likely to be published than studies that do not), both of which are related to precision.

Our simulation results indicate that regression-based extrapolation may suffer from bias in practice to the extent to which our small study effects mechanisms can be considered more or less plausible than the mechanisms assumed by regression-based extrapolation. If one believes that all these mechanisms are similarly plausible, our simulations then indicate that many plausible small study effects mechanisms may lead to substantial bias in regression-based extrapolation.

# Supplementary Tables

**Supplementary Table 1.** Performance of different meta-analysis estimators under scenario 1: zero true effect (i.e., $\beta=0$), no small study effects, and study sizes uniformly ranging from 100 to 5000 individuals.

| **Estimator** | **Statistic** | $\boldsymbol{K}$ **(**$\boldsymbol{I}^{\boldsymbol{2}}$**;** $\boldsymbol{\gamma}$**)** | | | |
| --- | --- | --- | --- | --- | --- |
|  |  | **5 (13.6%; 0.00)** | **10 (11.2%; 0.00)** | **30 (7.6%; 0.00)** | **50 (6.2%; 0.00)** |
| Fixed | Point estimate | 0.000 | 0.000 | 0.000 | 0.000 |
| Effects | Standard error | 0.018 | 0.013 | 0.007 | 0.006 |
|  | Coverage (%) | 95.2 | 94.9 | 94.9 | 95.4 |
|  | Rejection rate (%) | 4.8 | 5.1 | 5.1 | 4.6 |
| Regression- | Point estimate | 0.000 | 0.000 | 0.000 | 0.000 |
| based | Standard error | 0.071 | 0.044 | 0.024 | 0.018 |
| extrapolation | Coverage (%) | 86.0 | 91.3 | 93.3 | 93.9 |
|  | Rejection rate (%) | 14.0 | 8.7 | 6.7 | 6.1 |
| Trim-and-fill | Point estimate | 0.000 | 0.000 | 0.000 | 0.000 |
|  | Standard error | 0.021 | 0.014 | 0.008 | 0.006 |
|  | Coverage (%) | 94.1 | 94.0 | 92.0 | 91.7 |
|  | Rejection rate (%) | 6.0 | 6.0 | 8.0 | 8.3 |
| Weighted | Point estimate | 0.000 | 0.000 | 0.000 | 0.000 |
| Median | Standard error | 0.022 | 0.017 | 0.010 | 0.008 |
|  | Coverage (%) | 96.8 | 97.2 | 97.6 | 97.7 |
|  | Rejection rate (%) | 3.2 | 2.8 | 2.4 | 2.3 |
| MBE | Point estimate | 0.000 | 0.000 | 0.000 | 0.000 |
|  | Standard error | 0.028 | 0.023 | 0.017 | 0.015 |
|  | Coverage (%) | 98.1 | 98.7 | 99.3 | 99.5 |
|  | Rejection rate (%) | 1.9 | 1.3 | 0.7 | 0.5 |

$K$: number of studies.

$I^{2}$: between-study inconsistency.

$\gamma$: Egger test’s coefficient (i.e., slope in inverse variance weighted linear regression of effect estimates on standard errors).

MBE: mode-based estimate.

**Supplementary Table 2.** Between-study inconsistency ($I^{2}$) and funnel plot asymmetry ($\gamma$) according to the proportion of biased studies and number of studies ($K$) under scenario 2: zero true effect (i.e., $\beta=0$), small study effects through the bias term $b_{j}$, and study sizes uniformly ranging from 100 to 5000 individuals.

| **Statistic** | $\boldsymbol{K}$ | **Proportion (%) of biased studies (**$\boldsymbol{\delta}$ **parameter)** | | | | | | | | | | |
| --- | --- | --- | --- | --- | --- | --- | --- | --- | --- | --- | --- | --- |
|  |  | **0** | **10** | **20** | **30** | **40** | **50** | **60** | **70** | **80** | **90** | **100** |
| $I^{2}$ (%) | 5 | 13.5 | 38.7 | 56.7 | 69.7 | 78.2 | 83.1 | 86.3 | 87.2 | 87.3 | 85.9 | 83.5 |
|  | 10 | 11.3 | 49.9 | 70.2 | 80.7 | 86.0 | 88.8 | 89.7 | 90.3 | 90.1 | 89.5 | 88.0 |
|  | 30 | 7.9 | 64.5 | 80.3 | 86.1 | 88.6 | 90.0 | 90.7 | 91.0 | 90.9 | 90.4 | 89.5 |
|  | 50 | 6.4 | 68.1 | 81.7 | 86.6 | 88.9 | 90.2 | 90.8 | 91.1 | 91.0 | 90.5 | 89.6 |
| $\gamma$ | 5 | 0.00 | 1.01 | 1.99 | 3.02 | 3.93 | 4.74 | 5.91 | 6.85 | 7.75 | 8.85 | 9.67 |
|  | 10 | 0.00 | 0.85 | 1.71 | 2.65 | 3.47 | 4.41 | 5.31 | 6.19 | 6.99 | 7.86 | 8.77 |
|  | 30 | 0.00 | 0.82 | 1.66 | 2.47 | 3.28 | 4.11 | 4.93 | 5.73 | 6.57 | 7.40 | 8.21 |
|  | 50 | 0.00 | 0.81 | 1.62 | 2.44 | 3.26 | 4.06 | 4.88 | 5.69 | 6.51 | 7.31 | 8.11 |

$\gamma$: Egger test’s coefficient (i.e., slope in inverse variance weighted linear regression of effect estimates on standard errors).

**Supplementary Table 3.** Between-study inconsistency ($I^{2}$) and funnel plot asymmetry ($\gamma$) according to the minimum study size required for not being affected by publication bias ($N$) and number of studies ($K$) under scenario 3: zero true effect (i.e., $\beta=0$), small study effects through publication bias (assuming a linear relationship between $p_{j}$and $n_{j}$), and study sizes uniformly ranging from 100 to 5000 individuals.

| **Statistic** | $\boldsymbol{K}$ | $\boldsymbol{N}$ | | | | |
| --- | --- | --- | --- | --- | --- | --- |
|  |  | **1** | **1500** | **3000** | **4500** | **6000** |
| $I^{2}$ (%) | 5 | 13.5 | 15.9 | 18.9 | 17.3 | 14.5 |
|  | 10 | 11.4 | 14.1 | 16.4 | 15.1 | 11.2 |
|  | 30 | 7.7 | 10.8 | 14.8 | 12.5 | 7.2 |
|  | 50 | 6.4 | 9.7 | 13.9 | 11.4 | 5.5 |
| $\gamma$ | 5 | 0.00 | 1.06 | 2.12 | 2.53 | 2.52 |
|  | 10 | 0.00 | 1.21 | 2.18 | 2.53 | 2.54 |
|  | 30 | 0.00 | 1.33 | 2.22 | 2.55 | 2.55 |
|  | 50 | 0.00 | 1.33 | 2.22 | 2.55 | 2.56 |

$p_{j}$: maximum P-value allowed for publication for a study with $n_{j}$ participants. $\gamma$: Egger test’s coefficient (i.e., slope in inverse variance weighted linear regression of effect estimates on standard errors).

**Supplementary Table 4.** Between-study inconsistency ($I^{2}$) and funnel plot asymmetry ($\gamma$) according to the minimum study size required for not being affected by publication bias ($N$) and number of studies ($K$) under scenario 4: zero true effect (i.e., $\beta=0$), small study effects through publication bias (assuming a square root relationship between $p_{j}$and $n_{j}$), and study sizes uniformly ranging from 100 to 5000 individuals.

| **Statistic** | $\boldsymbol{K}$ | $\boldsymbol{N}$ | | | | |
| --- | --- | --- | --- | --- | --- | --- |
|  |  | **1** | **1500** | **3000** | **4500** | **6000** |
| $I^{2}$ (%) | 5 | 14.0 | 12.5 | 11.6 | 9.4 | 6.7 |
|  | 10 | 11.3 | 10.3 | 8.8 | 6.5 | 3.7 |
|  | 30 | 8.0 | 6.2 | 5.2 | 2.8 | 0.8 |
|  | 50 | 6.3 | 5.0 | 3.7 | 1.6 | 0.3 |
| $\gamma$ | 5 | 0.00 | 0.68 | 1.31 | 1.59 | 1.62 |
|  | 10 | 0.00 | 0.77 | 1.35 | 1.62 | 1.63 |
|  | 30 | 0.00 | 0.82 | 1.38 | 1.63 | 1.64 |
|  | 50 | 0.00 | 0.83 | 1.39 | 1.63 | 1.65 |

$p_{j}$: maximum P-value allowed for publication for a study with $n_{j}$ participants. $\gamma$: Egger test’s coefficient (i.e., slope in inverse variance weighted linear regression of effect estimates on standard errors).

**Supplementary Table 5.** Between-study inconsistency ($I^{2}$) and funnel plot asymmetry ($\gamma$) according to the minimum study size required for not being affected by publication bias ($N$) and number of studies ($K$) under scenario 5: zero true effect (i.e., $\beta=0$), small study effects through publication bias (assuming a quadratic relationship between $p_{j}$and $n_{j}$), and study sizes uniformly ranging from 100 to 5000 individuals.

| **Statistic** | $\boldsymbol{K}$ | $\boldsymbol{N}$ | | | | |
| --- | --- | --- | --- | --- | --- | --- |
|  |  | **1** | **1500** | **3000** | **4500** | **6000** |
| $I^{2}$ (%) | 5 | 13.3 | 26.0 | 36.1 | 37.0 | 34.0 |
|  | 10 | 11.5 | 25.4 | 38.6 | 40.4 | 34.9 |
|  | 30 | 7.6 | 25.8 | 41.6 | 43.5 | 37.9 |
|  | 50 | 6.5 | 26.1 | 42.5 | 44.7 | 39.2 |
| $\gamma$ | 5 | 0.00 | 1.67 | 3.26 | 3.88 | 3.82 |
|  | 10 | 0.00 | 1.91 | 3.37 | 3.90 | 3.84 |
|  | 30 | 0.00 | 2.06 | 3.43 | 3.89 | 3.86 |
|  | 50 | 0.00 | 2.08 | 3.43 | 3.90 | 3.86 |

$p_{j}$: maximum P-value allowed for publication for a study with $n_{j}$ participants. $\gamma$: Egger test’s coefficient (i.e., slope in inverse variance weighted linear regression of effect estimates on standard errors).

**Supplementary Table 6.** Between-study inconsistency ($I^{2}$) and funnel plot asymmetry ($\gamma$) according to $N_{small}$, $N_{large}$, and number of studies ($K$) under scenario 6: zero true effect (i.e., $\beta=0$), small study effects through publication bias (assuming a step function relationship between $p_{j}$and $n_{j}$), and study sizes uniformly ranging from 100 to 5000 individuals.

| **Statistic** | $\boldsymbol{K}$ | $\boldsymbol{N}_{\boldsymbol{small}}\boldsymbol{;}\boldsymbol{N}_{\boldsymbol{large}}$ | | | |
| --- | --- | --- | --- | --- | --- |
|  |  | **0 ; 0** | **500 ; 1000** | **1000 ; 2000** | **2000 ; 4000** |
| $I^{2}$ (%) | 5 | 13.7 | 26.2 | 38.3 | 53.1 |
|  | 10 | 11.5 | 25.4 | 40.2 | 56.3 |
|  | 30 | 7.9 | 25.1 | 42.4 | 58.7 |
|  | 50 | 6.3 | 25.4 | 43.4 | 59.1 |
| $\gamma$ | 5 | 0.00 | 0.89 | 2.05 | 3.95 |
|  | 10 | 0.00 | 1.09 | 2.29 | 3.98 |
|  | 30 | 0.00 | 1.26 | 2.46 | 3.91 |
|  | 50 | 0.00 | 1.29 | 2.49 | 3.89 |

$p_{j}$: maximum P-value allowed for publication for a study with $n_{j}$ participants. $N_{small}$: maximum sample size for a study to be classified as small. $N_{large}$: minimum sample size for a study to be classified as large. $\gamma$: Egger test’s coefficient (i.e., slope in inverse variance weighted linear regression of effect estimates on standard errors).

**Supplementary Table 7.** Performance of different meta-analysis estimators under scenario 7: true effect $\beta=0$.02, no small study effects, and study sizes uniformly ranging from $n_{1}$ to $n_{2}$.

| **Estimator** | **Statistic** | $\boldsymbol{K}$ **(**$\boldsymbol{I}^{\boldsymbol{2}}$**;** $\boldsymbol{\gamma}$**)** | | | | | | | | |
| --- | --- | --- | --- | --- | --- | --- | --- | --- | --- | --- |
|  |  | **5 (13.3%; 0.00)** | **10 (11.4%; 0.00)** | **30 (7.7%; 0.00)** | **50 (6.4%; 0.00)** | **5 (13.6%; 0.00)** | | **10 (11.5%; 0.00)** | **30 (7.7%; 0.00)** | **50 (6.3%; 0.00)** |
|  |  | $\boldsymbol{n}_{\boldsymbol{1}}\boldsymbol{=100;}\boldsymbol{n}_{\boldsymbol{2}}\boldsymbol{=1000}$ | | | | | $\boldsymbol{n}_{\boldsymbol{1}}\boldsymbol{=100}\boldsymbol{0;}\boldsymbol{n}_{\boldsymbol{2}}\boldsymbol{=5000}$ | | | |
| Fixed | Point estimate | 0.021 | 0.020 | 0.020 | 0.020 | 0.020 | | 0.020 | 0.020 | 0.020 |
| Effects | Standard error | 0.039 | 0.027 | 0.016 | 0.012 | 0.017 | | 0.012 | 0.007 | 0.005 |
|  | Coverage (%) | 95.2 | 95.2 | 94.9 | 94.6 | 94.7 | | 95.1 | 94.5 | 95.2 |
|  | Rejection rate (%) | 8.6 | 11.5 | 25.7 | 38.6 | 22.9 | | 41.1 | 85.0 | 97.1 |
| Regression- | Point estimate | 0.021 | 0.021 | 0.020 | 0.020 | 0.019 | | 0.020 | 0.020 | 0.020 |
| based | Standard error | 0.184 | 0.114 | 0.063 | 0.048 | 0.099 | | 0.062 | 0.034 | 0.026 |
| extrapolation | Coverage (%) | 85.6 | 91.6 | 93.8 | 94.4 | 85.7 | | 91.7 | 93.7 | 94.2 |
|  | Rejection rate (%) | 14.2 | 8.6 | 7.5 | 7.5 | 15.1 | | 9.7 | 10.3 | 13.3 |
| Trim-and-fill | Point estimate | 0.021 | 0.020 | 0.020 | 0.020 | 0.020 | | 0.020 | 0.020 | 0.020 |
|  | Standard error | 0.043 | 0.030 | 0.017 | 0.013 | 0.018 | | 0.013 | 0.007 | 0.006 |
|  | Coverage (%) | 94.3 | 93.5 | 89.9 | 88.6 | 93.7 | | 93.1 | 88.0 | 84.6 |
|  | Rejection rate (%) | 8.9 | 12.0 | 26.8 | 38.1 | 20.8 | | 37.2 | 75.4 | 88.6 |
| Weighted | Point estimate | 0.021 | 0.020 | 0.020 | 0.020 | 0.020 | | 0.020 | 0.020 | 0.020 |
| Median | Standard error | 0.048 | 0.036 | 0.022 | 0.017 | 0.021 | | 0.016 | 0.009 | 0.007 |
|  | Coverage (%) | 96.3 | 97.1 | 97.6 | 97.3 | 96.6 | | 97.2 | 97.3 | 97.6 |
|  | Rejection rate (%) | 5.5 | 6.0 | 11.6 | 18.1 | 14.6 | | 23.5 | 56.9 | 80.8 |
| MBE | Point estimate | 0.020 | 0.021 | 0.021 | 0.020 | 0.020 | | 0.020 | 0.020 | 0.020 |
|  | Standard error | 0.063 | 0.053 | 0.040 | 0.035 | 0.028 | | 0.024 | 0.018 | 0.016 |
|  | Coverage (%) | 98.3 | 99.0 | 99.6 | 99.7 | 98.5 | | 99.2 | 99.7 | 99.8 |
|  | Rejection rate (%) | 2.9 | 2.0 | 2.5 | 2.8 | 6.6 | | 7.7 | 13.8 | 18.7 |

$K$: number of studies.

$I^{2}$: between-study inconsistency.

$\gamma$: Egger test’s coefficient (i.e., slope in inverse variance weighted linear regression of effect estimates on standard errors).

MBE: mode-based estimate.

# Supplementary Figures

**Supplementary Figure 1. Illustration of regression-based extrapolation (panels A and C) and trim-and-fill (panels B and C) using the catheter (panels A and B) and aspirin (panels C and D) datasets.**


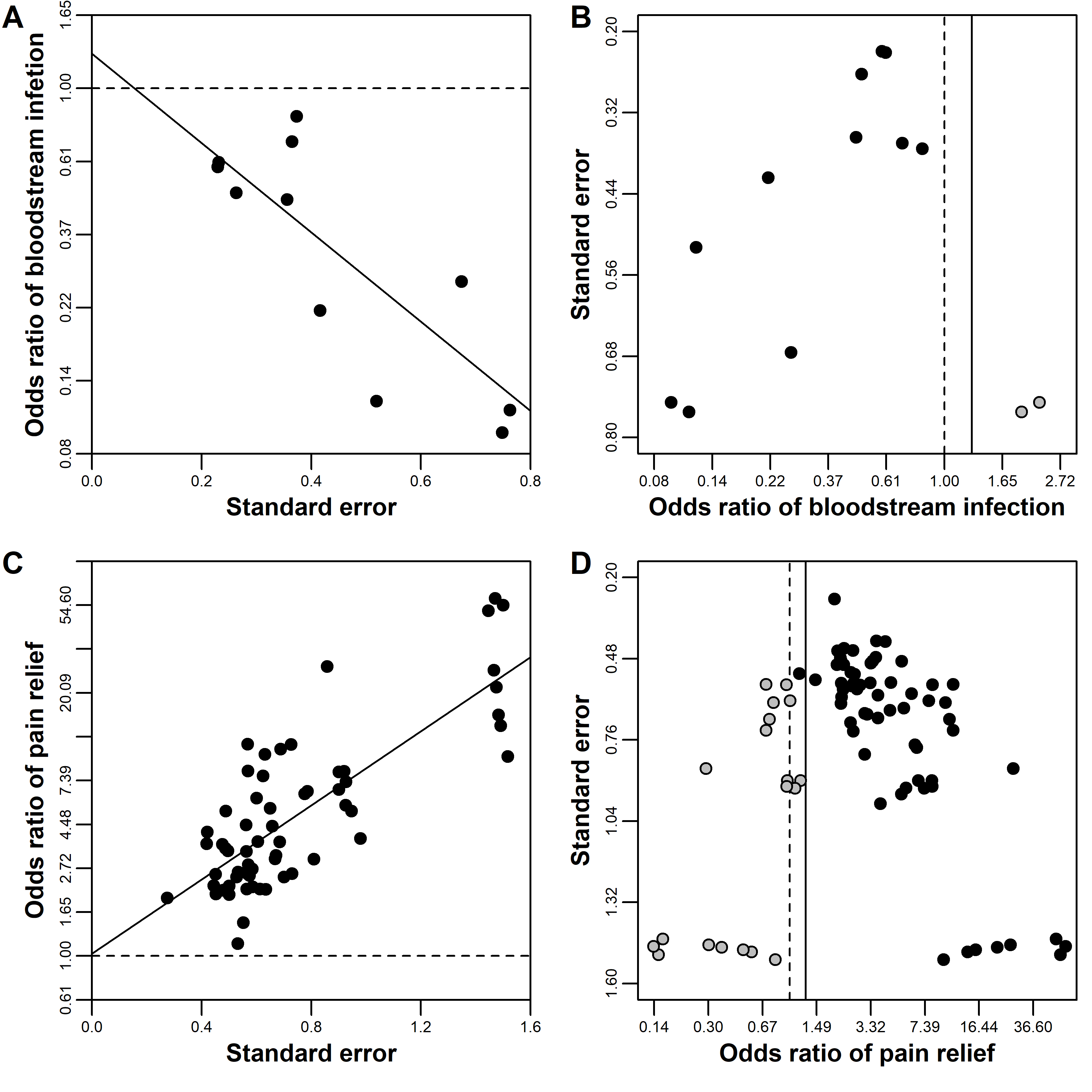


Dashed line passes through 1. In panels A and C, the solid line is the fitted regression line. The y-intercept (i.e., the y-coordinate where the fitted line crosses the y-axis) is the estimated effect in a study of infinite size. This intercept is shown as a solid line in panels B and D, where observed studies are shown in black, and data points generated via data augmentation are shown in grey.

**Supplementary Figure 2. Illustration of the functional relationships between** $\boldsymbol{p}_{\boldsymbol{j}}$**and** $\boldsymbol{n}_{\boldsymbol{j}}$ **induced by different publication bias mechanisms.**

**
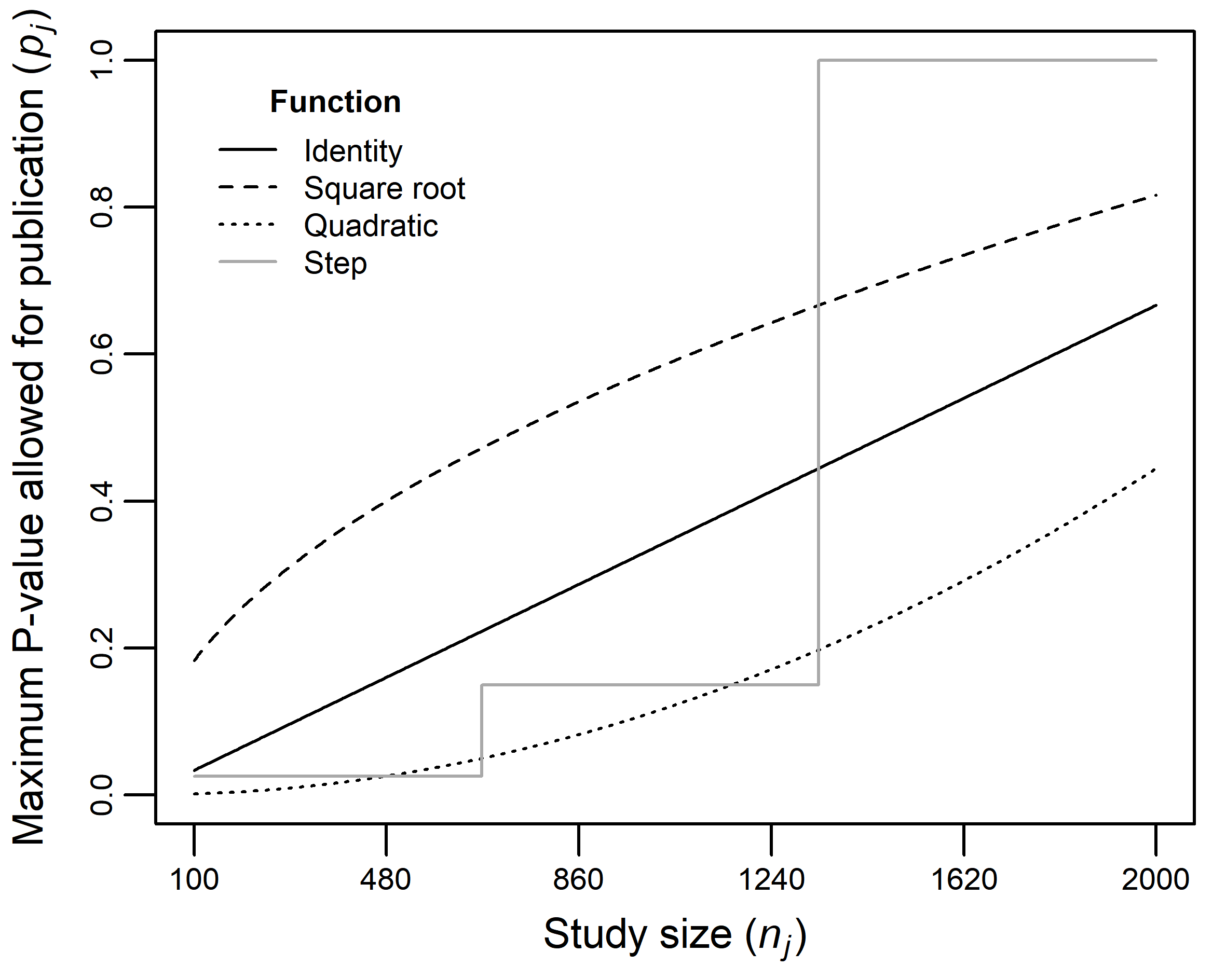
**

$p_{j}$: maximum P-value allowed for publication for a study with $n_{j}$ participants.

**Supplementary Figure 3. Bias (solid lines) and coverage (dashed lines) of the fixed effects (black), regression-based extrapolation (red), trim-and-fill (green), weighted median (dark blue) and mode-based estimate (light blue) under scenario 1: zero true effect (i.e.,** $\boldsymbol{\beta=0}$**), no small study effects, and study sizes uniformly ranging from 100 to 5000 individuals.**


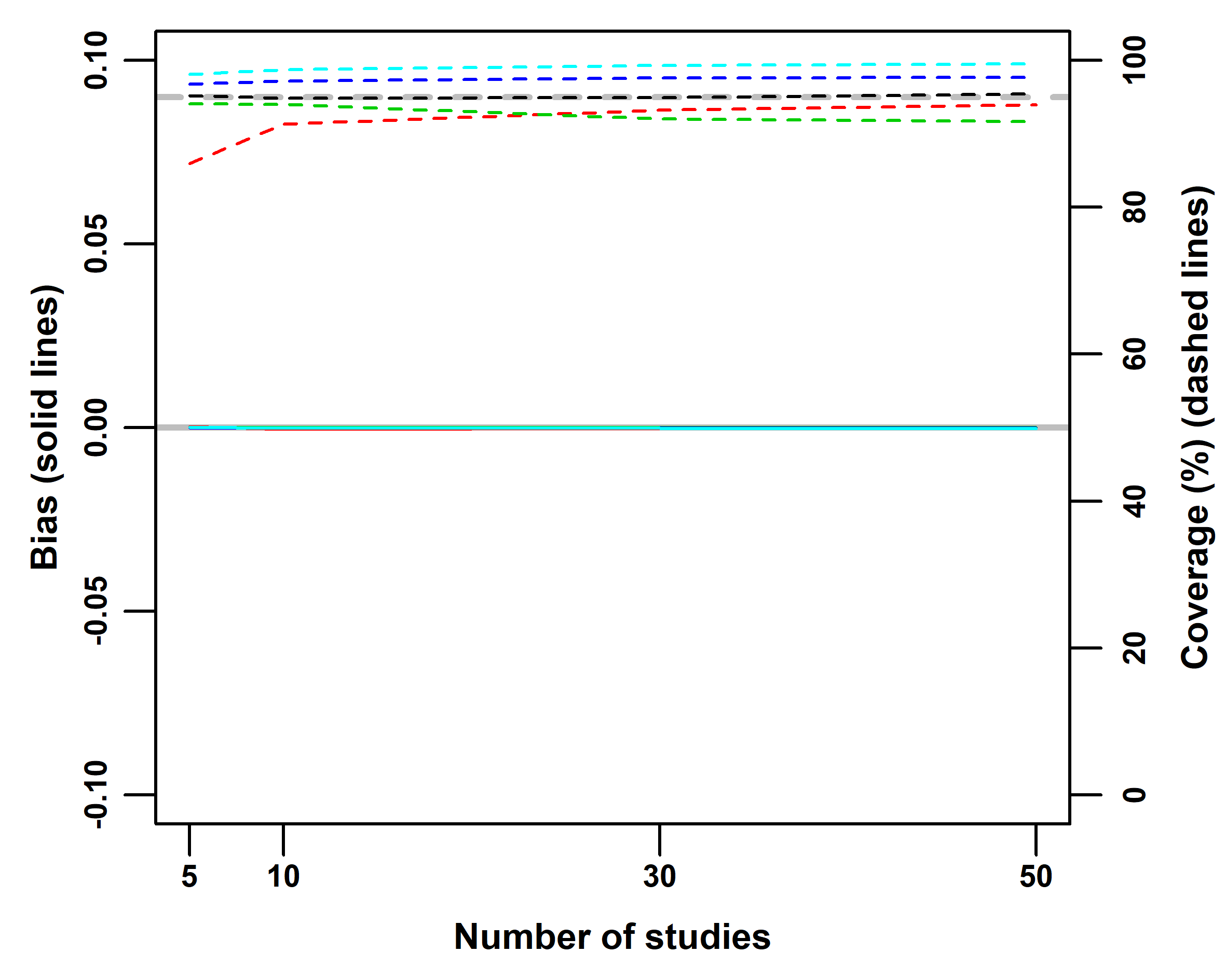


The grey solid line indicates zero bias. The gray dashed line indicates 95% coverage.

**Supplementary Figure 4. Bias (solid lines) and coverage (dashed lines) of the fixed effects (black), regression-based extrapolation (red), trim-and-fill (green), weighted median (dark blue) and mode-based estimate (light blue) under scenario 4: zero true effect (i.e.,** $\boldsymbol{\beta=0}$**), small study effects through publication bias (assuming a square root relationship between** $\boldsymbol{p}_{\boldsymbol{j}}$**and** $\boldsymbol{n}_{\boldsymbol{j}}$**), and study sizes uniformly ranging from 100 to 5000 individuals.**


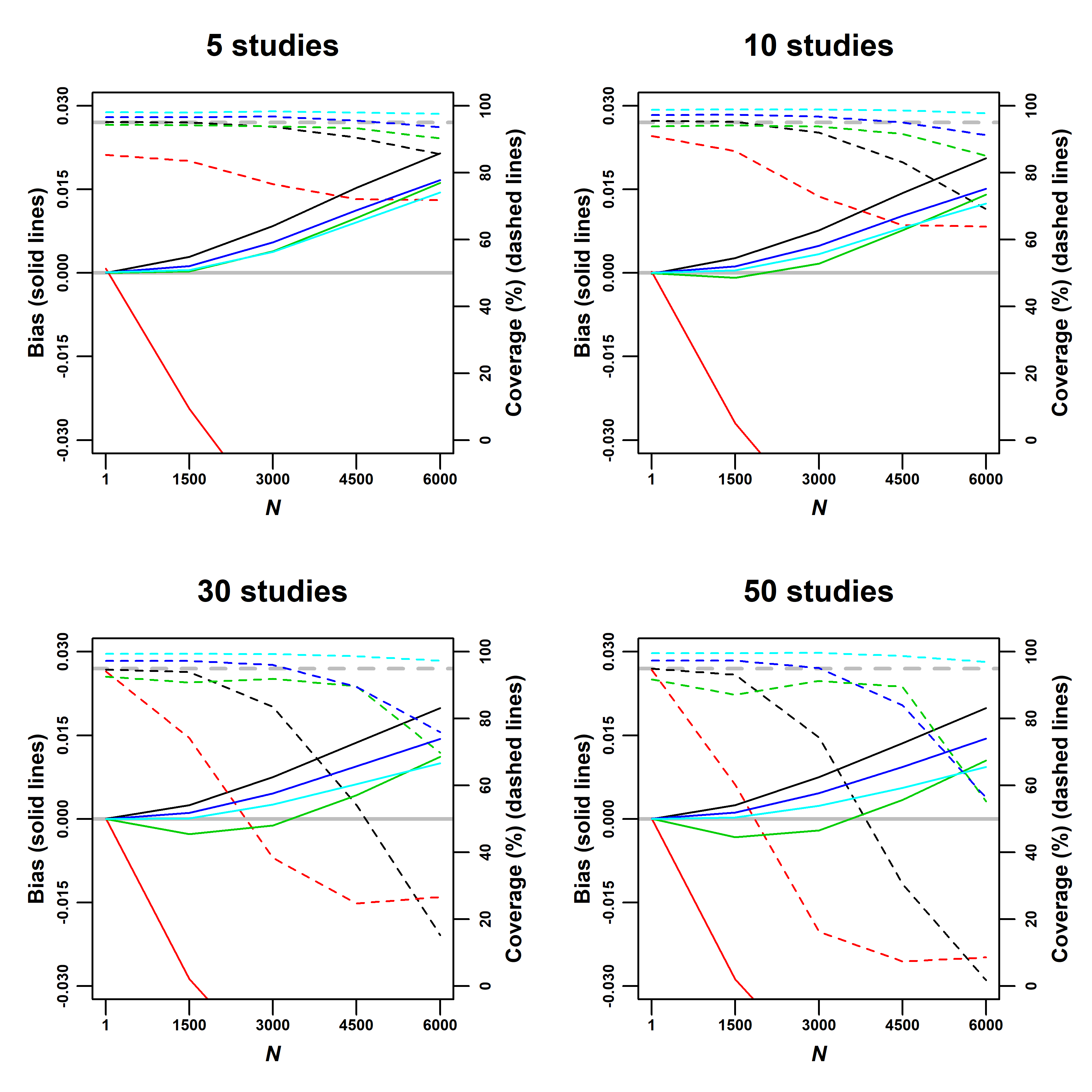


$p_{j}$: maximum P-value allowed for publication for a study with $n_{j}$ participants. $N$: study size threshold, with studies larger than or equally sized to $n^{*}$ not being affected by small study effects.

The grey line indicates zero bias. The gray dashed line indicates 95% coverage.

**Supplementary Figure 5. Bias (solid lines) and coverage (dashed lines) of the fixed effects (black), regression-based extrapolation (red), trim-and-fill (green), weighted median (dark blue) and mode-based estimate (light blue) under scenario 5: zero true effect (i.e.,** $\boldsymbol{\beta=0}$**), small study effects through publication bias (assuming a quadratic relationship between** $\boldsymbol{p}_{\boldsymbol{j}}$**and** $\boldsymbol{n}_{\boldsymbol{j}}$**), and study sizes uniformly ranging from 100 to 5000 individuals.**


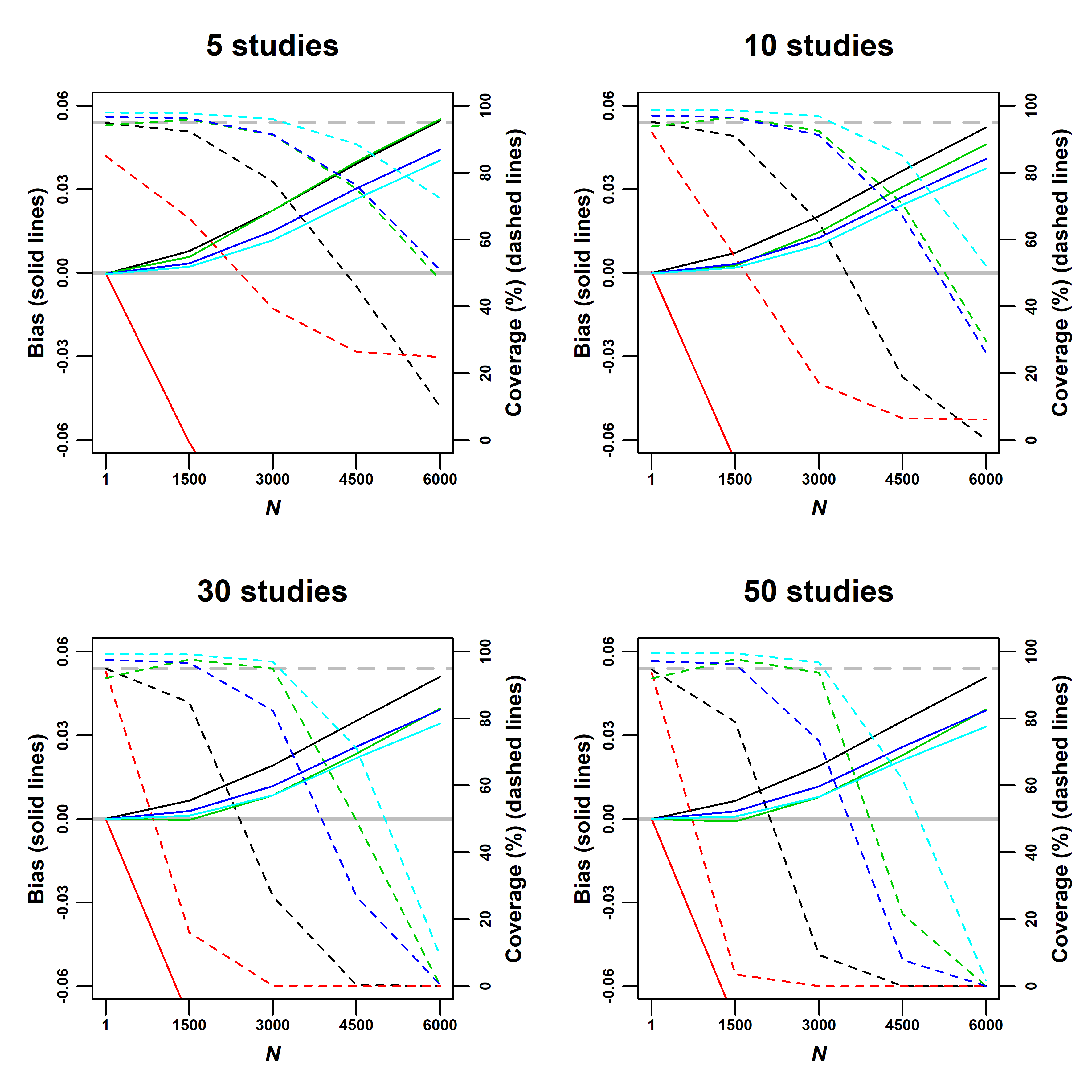


$p_{j}$: maximum P-value allowed for publication for a study with $n_{j}$ participants. $N$: study size threshold, with studies larger than or equally sized to $n^{*}$ not being affected by small study effects.

The grey line indicates zero bias. The gray dashed line indicates 95% coverage.

**Supplementary Figure 6. Bias (solid lines) and coverage (dashed lines) of the fixed effects (black), regression-based extrapolation (red), trim-and-fill (green), weighted median (dark blue) and mode-based estimate (light blue) under scenario 6: zero true effect (i.e.,** $\boldsymbol{\beta=0}$**), small study effects through publication bias (assuming a step function relationship between** $\boldsymbol{p}_{\boldsymbol{j}}$**and** $\boldsymbol{n}_{\boldsymbol{j}}$**), and study sizes uniformly ranging from 100 to 5000 individuals.**


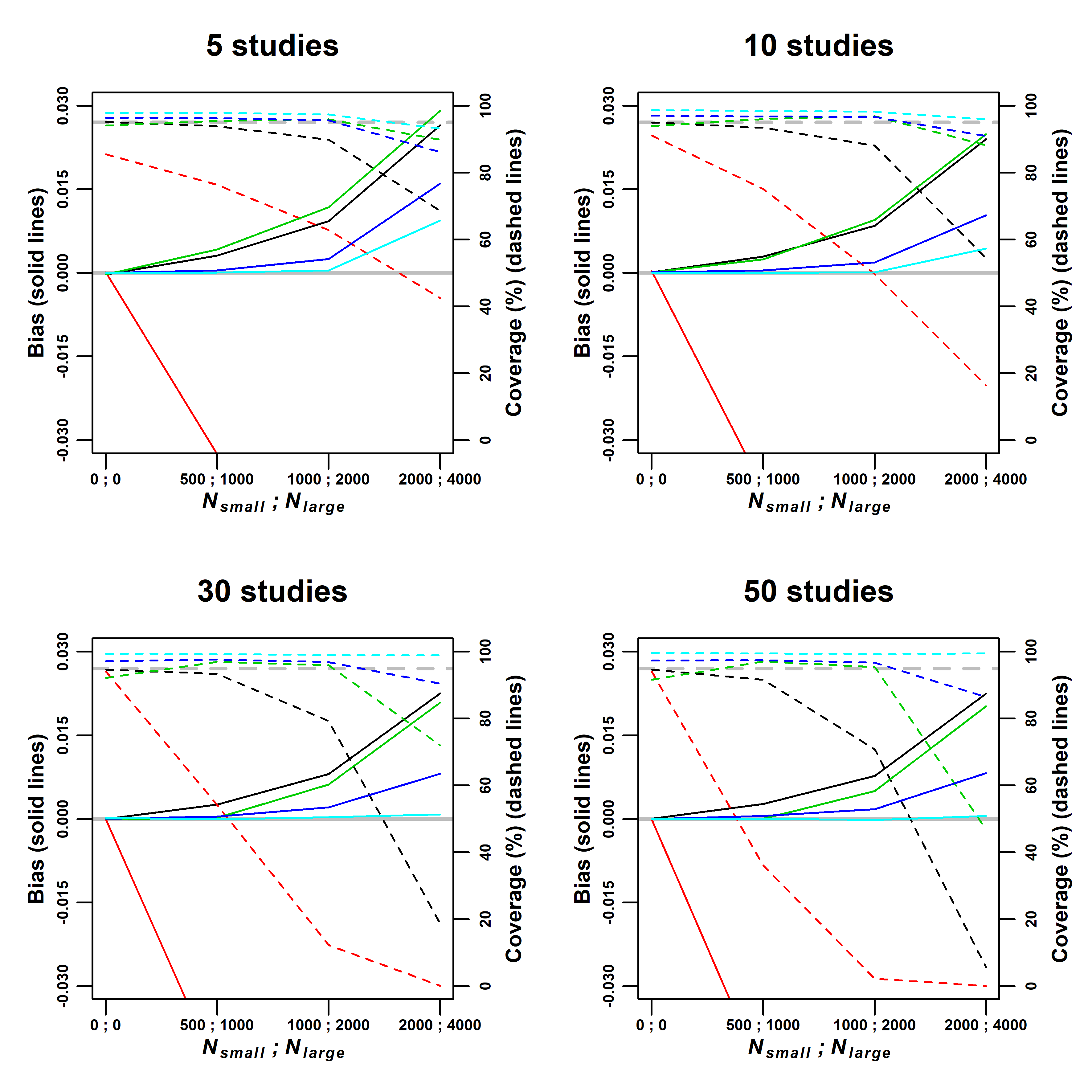


$p_{j}$: maximum P-value allowed for publication for a study with $n_{j}$ participants. $N_{small}$: minimum sample size for a study to be classified as medium-sized. $N_{large}$:minimum sample size for a study to be classified as large.

The grey line indicates zero bias. The gray dashed line indicates 95% coverage.

**Supplementary Figure 7. Rejection rate (solid lines) and coverage (dashed lines) of the fixed effects (black), regression-based extrapolation (red), trim-and-fill (green), weighted median (dark blue) and mode-based estimate (light blue) under scenario 1: true effect** $\boldsymbol{\beta=0.02}$**, no small study effects, and study sizes uniformly ranging from** $\boldsymbol{n}_{\boldsymbol{1}}$ **to** $\boldsymbol{n}_{\boldsymbol{2}}$ **individuals.**


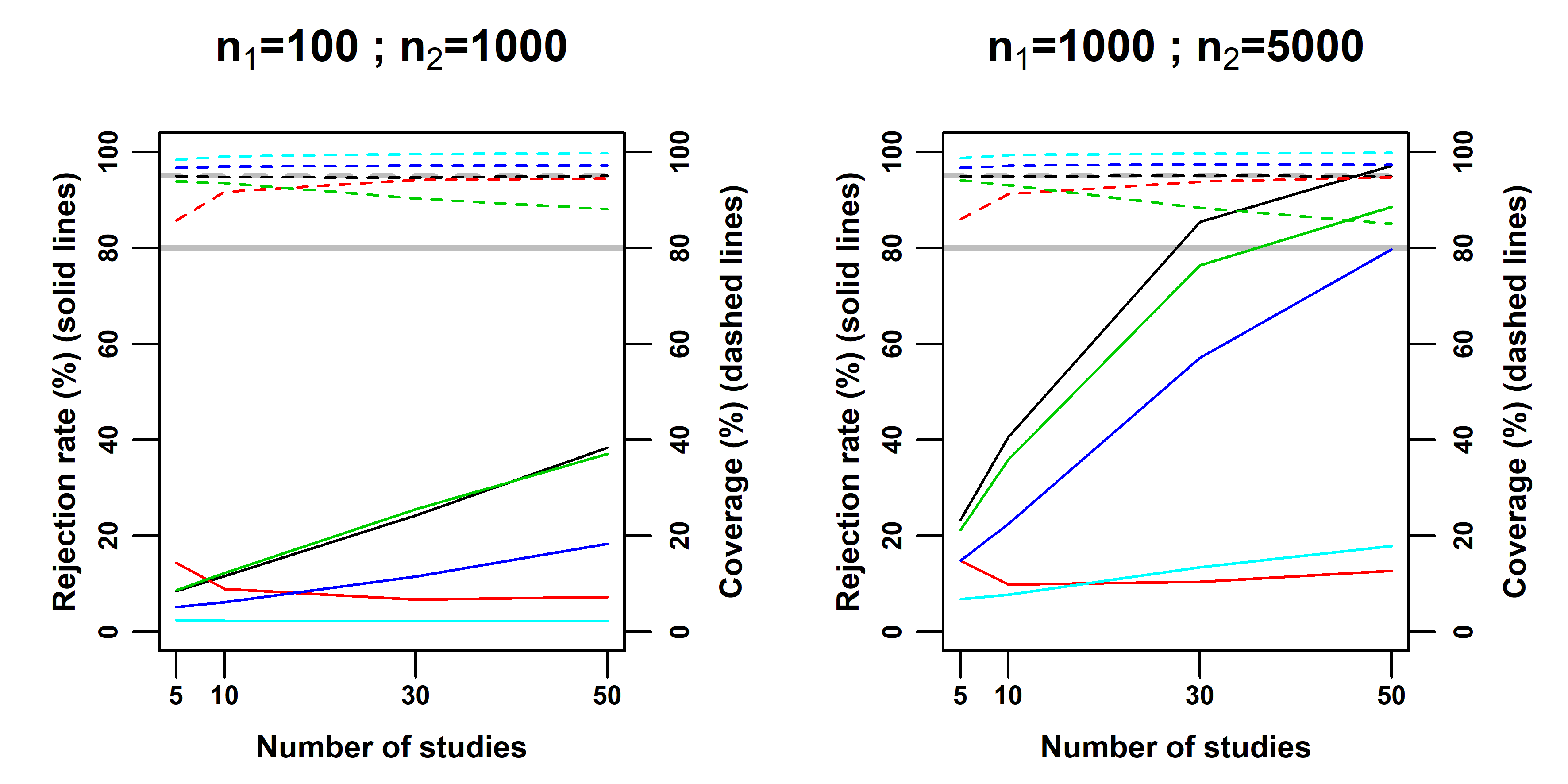


The grey solid line indicates 80% rejection rate. The gray dashed line indicates 95% coverage.

**Supplementary Figure 8. Illustration of the relationship between bias and standard error induced by different small study effects mechanisms.**


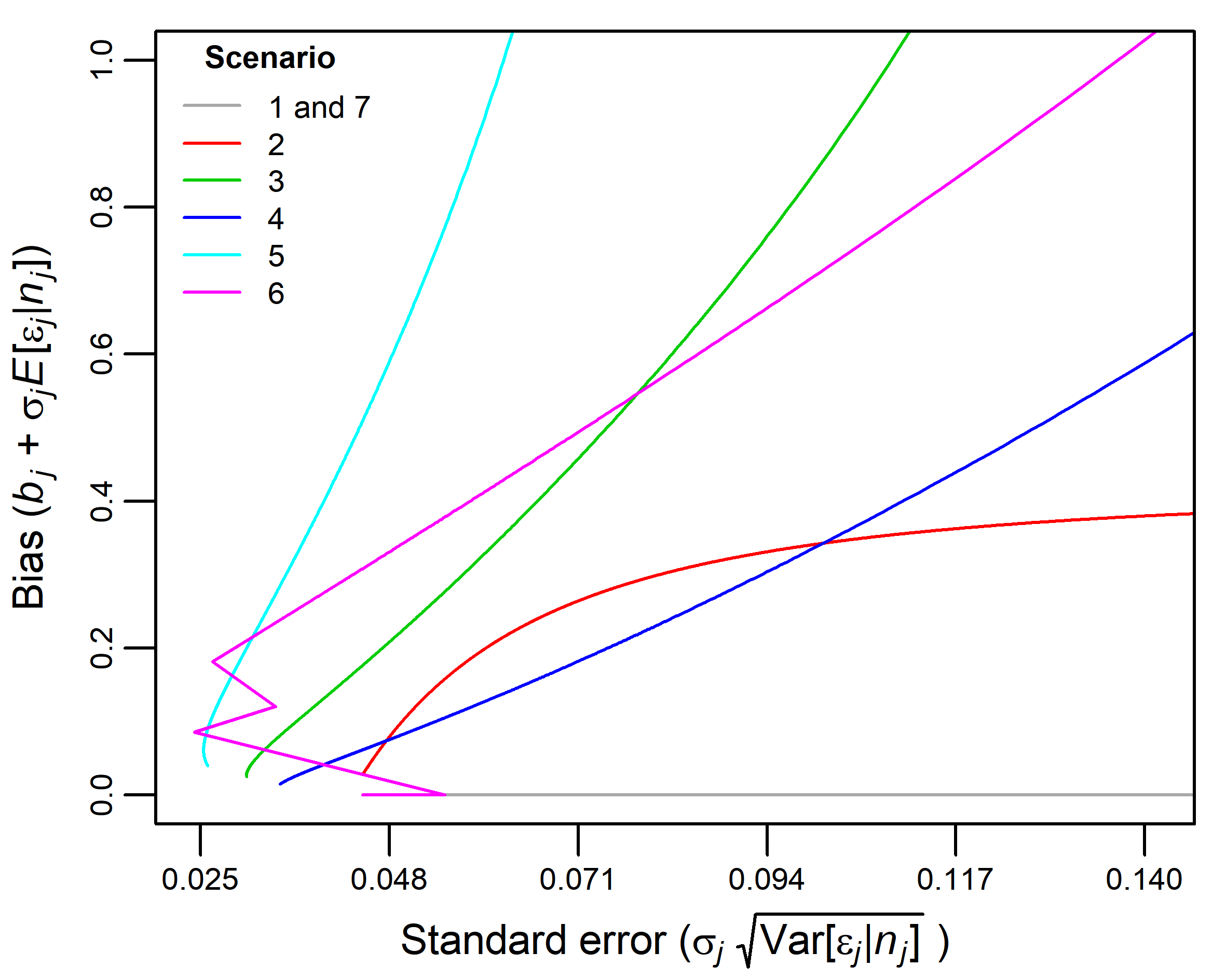


# Software code (R language)

## Weighted median

#beta.in: point estimates (mean differences, log-odds ratio, etc.).

#se.in: standard errors

#alpha: confidence level of the confidence intervals. Defaults to 0.05 (i.e., 95% confidence intervals)

#n_boot: number of bootstrap iterations. Defaults to 1e4 (i.e., 10,000 iterations).

WeightedMedianMeta <- function(beta.in, se.in, alpha=0.05,n_boot=1e4) {

#Function to calculate the point estimate

weighted.median <- function(beta.in, weights.in) {

beta.order <- beta.in[order(beta.in)]

weights.order <- weights.in[order(beta.in)]

weights.sum <- cumsum(weights.order)-0.5*weights.order

weights.sum <- weights.sum/sum(weights.order)

below <- max(which(weights.sum<0.5))

weighted.median.est <- beta.order[below] + (beta.order[below+1]-beta.order[below])*

(0.5-weights.sum[below])/(weights.sum[below+1]-weights.sum[below])

return(weighted.median.est)

}

#Calculate point estimate

weights <- se.in^-2 #Inverse-variance weights

combined.beta <- weighted.median(beta.in, weights) #Inverse-variance weighted median

#Calculate standard errors through bootstrapping

boot.dist <- numeric(n_boot)

for(a in 1:n_boot) {

beta.boot <- rnorm(n=length(beta.in), mean=beta.in, sd=se.in)

boot.dist[a] <- weighted.median(beta.boot, weights)

}

combined.se <- mad(boot.dist)

#Calculate confidence intervals

ci <- combined.beta+c(-1,1)*qnorm(1-alpha/2)*combined.se

#Calculate P-value

P <- pnorm(abs(combined.beta)/combined.se, lower.tail=F)*2

#Provide results

results <- c(combined.beta, combined.se, ci, P)

names(results) <- c('Beta', 'SE', 'CIlow', 'CIupp', 'Pvalue')

return(results)

}

## Mode-based estimate

#beta.in: point estimates (mean differences, log-odds ratio, etc.).

#se.in: standard errors

#alpha: confidence level of the confidence intervals. Defaults to 0.05 (i.e., 95% confidence intervals)

#n_boot: number of bootstrap iterations. Defaults to 1e4 (i.e., 10,000 iterations).

MBEMeta <- function(beta.in, se.in, alpha=0.05, n_boot=1e4) {

#Function to calculate the point estimate

MBE <- function(beta.in, weights.in) {

bw <- 0.9*(min(sd(beta.in), mad(beta.in)))/length(beta.in)^(1/5) #Bandwidth

weights <- weights.in/sum(weights.in) #Standardising weights

EDF <- density(beta.in, bw=bw, weights=weights) #Weighted empirical density function

MBE.est <- EDF$x[which.max(EDF$y)] #Calculate point estimate

return(MBE.est)

}

#Calculate point estimate

weights <- se.in^-2 #Inverse-variance weights

combined.beta <- MBE(beta.in, weights) #Inverse-variance weighted MBE

#Calculate standard errors through bootstrapping

boot.dist <- numeric(n_boot)

for(a in 1:n_boot) {

beta.boot <- rnorm(n=length(beta.in), mean=beta.in, sd=se.in)

boot.dist[a] <- MBE(beta.boot, weights)

}

combined.se <- mad(boot.dist)

#Calculate confidence intervals

ci <- combined.beta+c(-1,1)*qnorm(1-alpha/2)*combined.se

#CalculateP-value

P <- pnorm(abs(combined.beta)/combined.se, lower.tail=F)*2

#Provide results

results <- c(combined.beta, combined.se, ci, P)

names(results) <- c('Beta', 'SE', 'CIlow', 'CIupp', 'Pvalue')

return(results)

}
